# Doxorubicin Conjugated to Immunomodulatory Anticancer Lactoferrin Displays Improved Cytotoxicity Overcoming Prostate Cancer Chemo resistance and Inhibits Tumour Development in TRAMP Mice

**DOI:** 10.1038/srep32062

**Published:** 2016-08-31

**Authors:** Jayanth Suryanarayanan Shankaranarayanan, Jagat R. Kanwar, Afrah Jalil Abd AL-Juhaishi, Rupinder K. Kanwar

**Affiliations:** 1 Nanomedicine-Laboratory of Immunology and Molecular Biomedical Research, School of Medicine, Faculty of Health, C-MMR, Deakin University, Geelong, Victoria 3216, Australia

## Abstract

Advanced, metastatic, castration resistant and chemo-resistant prostate cancer has triggered change in the drug development landscape against prostate cancer. Bovine lactoferrin (bLf) is currently attracting attention in clinics for its anti-cancer properties and proven safety profile. bLf internalises into cancer cells via receptor mediated endocytosis, boosts immunity and complements chemotherapy. We employed bLf as an excellent functional carrier protein for delivering doxorubicin (Dox) into DU145 cells, CD44+/EpCAM+ double positive enriched DU145 3D prostaspheres and drug resistant ADR1000-DU145 cells, thus circumventing Dox efflux, to overcome chemo-resistance. Successful bLf-Dox conjugation with iron free or iron saturated bLf forms did not affect the integrity and functionality of bLf and Dox. bLf-Dox internalised into DU145 cells within 6 h, enhanced nuclear Dox retention up to 24 h, and proved significantly effective (p < 0.001) in reducing LC_50_ value of Dox from 5.3 μM to 1.3 μM (4 fold). Orally fed iron saturated bLf-Dox inhibited tumour development, prolonged survival, reduced Dox induced general toxicity, cardiotoxicity, neurotoxicity in TRAMP mice and upregulated serum levels of anti-cancer molecules TNF-α, IFN-γ, CCL4 and CCL17. The study identifies promising potential of a novel and safer bLf-Dox conjugate containing a conventional cytotoxic drug along with bLf protein to target drug resistance.

Prostate cancer is one of the few cancers where chemotherapy is not the primary mode of therapy and is used only when surgery and androgen ablation therapy fails. Also, castration therapy is increasingly resulting in the emergence of hormone-insensitive and highly chemo-resistant tumour cells^1^ which limits the use of cytotoxic drugs in prostate cancer. Hence, it is the need of the hour to develop effective chemotherapeutics that can cause greater cancer specific cell death and overcome chemo-resistance at lower doses.

Doxorubicin (Dox) -the most frequently used chemotherapeutic targets actively dividing cells by intercalating with the nuclear DNA and preventing the activity of human topoisomerase II enzyme^2^. Earlier Dox used to be the primary highly effective mode of therapy to prostate cancer^3^ however, the increased risk of cardiac arrests due to cardio-toxicity of Dox and the chemo-resistance offered by prostate cancer, reduced the use of Dox^4^. Dox has consistently performed as an efficient chemotherapeutic in cell culture, however several combination strategies^5^ have been employed to improve its *in vivo* efficiency^6^. Although these strategies were able to overcome the non-specific cardio toxicity of Dox, they failed to overcome the chemo-resistance conferred due to the activation of several drug resistance proteins such as P-glycoprotein (P-gp) upon Dox exposure.

Apart from P-gp, several other molecules have been found to play a role in prostate cancer chemo-resistance^7^ such as multidrug resistance related protein 1 (MRP-1), which plays a greater role than P-gp in prostate cancer^8^. Anti-apoptotic protein Bcl-2, as a mediator of chemo-resistance and hormonal resistance, was established in prostate cancer^9^ along with another key molecular player PTEN which is often mutated or suppressed in case of advanced prostate cancer^10^.

Survivin plays a pivotal role in many pathways relating to therapeutic resistance generally in tumours including prostate cancer^11^,^12^ both *in vitro and in vivo*^13^. Clonally derived prostate cancer stem like cells (CSCs) expressing high levels of CD44, CD133 and nestin from patient tissues were able to form highly drug resistant prostate tumours *in vivo*^14^. These studies suggest that prostate cancer chemo-resistance can be overcome by targeting the crucial molecular regulators such as survivin; MRP-1, Bcl-2 and PTEN along with CSCs in combination to Dox.

Antibody-drug conjugates have been the focus of many anti-cancer studies to improve the specificity of Dox and overcome chemo-resistance^15^. A recent strategy of delivering Dox, targeted to mitochondria by a mitochondrial localising peptide, improved on the drug resistance response of cells to Dox^16^. However, these strategies only work by means of physical constraint and does not regulate the molecular activation of drug resistance pathway, hence a need for conjugating Dox to a biologically active molecule was considered.

Bovine lactoferrin (bLf) is an iron binding milk glycoprotein, investigated extensively by our laboratory and others for its anti-inflammatory, immuno-modulatory^17^, anti-microbial^18^, anti-oxidant^19^ and proven anti-cancer properties^20^. The commercially available native form of bovine lactoferrin (Nat-bLf) and the iron free form of bLf (Apo-bLf) have been found to have interestingly different anti-cancer properties^21^,^22^. Further we have reported anti-survivin activity against colon CSCs and tumours by nanoformulated iron saturated form of bLf (Fe-bLf)^23^.

Interestingly, bLf is one of the easiest proteins for EDC-NHS based conjugation, due to its high chemical and temperature stability and has been used in a number of studies for the conjugation of PEG-NHS esters^24^. Engineered nanoparticles containing bLf to conjugated PEG-PLA block copolymer are used as targeting moiety for the uptake of these nanoparticles by the transferrin receptor in the brain cells^25^. Deferasirox - bLf conjugates developed using EDC-NHS coupling reaction showed effective uptake of the drug by the PC-12 brain cells, and in rat models when compared to the non-conjugated deferasirox alone^26^. These studies demonstrated the potential of bLf as a carrier and targeting protein, a less explored facet of this multi-dimensional protein.

Earlier we have shown that orally fed Fe-bLf act as a fortifying agent for augmenting cancer chemotherapy in mice, and sensitizes drug resistant tumours to widely employed chemotherapeutics including Dox^27^. Our recent preclinical studies revealed that both Apo-bLf and Fe-bLf rapidly internalise into cells mediated by lactoferrin, transferrin and lipo-protein receptor related proteins (LRP) receptors^23^,^28^. Considering, the ability of bLf (in iron free and iron saturated) forms to overcome chemo-resistance and to internalise rapidly in cancer cells, two protein-drug conjugates of Apo-bLf and Fe-bLf with Dox were prepared and their ability to overcome drug efflux mechanism was investigated.

## Results

### Synthesis of bLf-Dox conjugates

**Figure 1:**
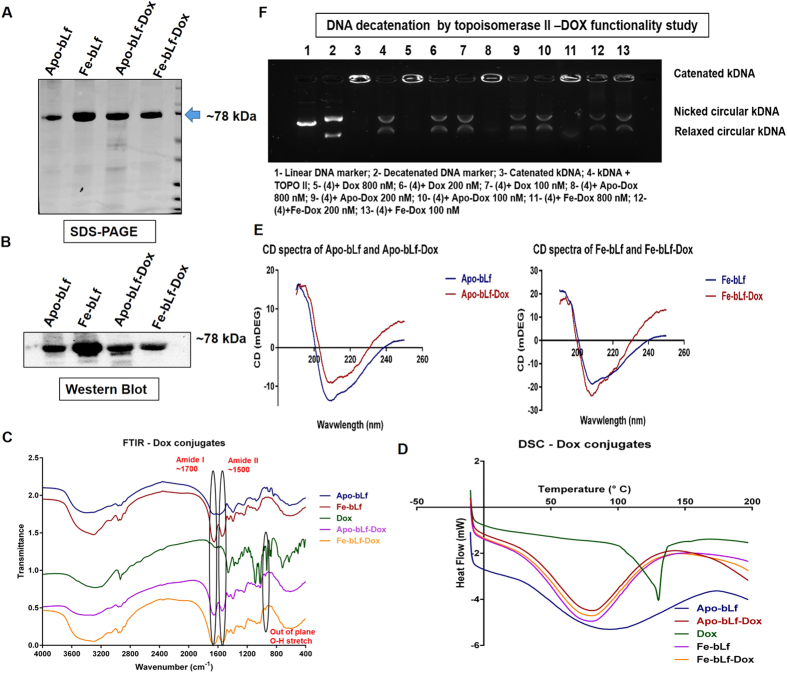
Synthesis and characterisation of bLf-Dox conjugates. (**A**) Image indicates the purity of synthesised forms of bLf-Dox conjugates prepared from Apo-bLf and Fe-bLf using SDS PAGE represented by a single non-denatured band around 78 kDa. (**B**) Western blot was carried out for the synthesised bLf-Dox conjugates against goat anti-bLf monoclonal antibody (1:1000) showing antigen reactivity ~78 kDa. (**C**) FTIR spectroscopy analysis was performed between 4000 and 400 cm^−1^ at a resolution of 4 cm^−1^ averaging 10 scans. The spectrum of each sample was then plotted with percentage transmittance against wavenumber. Crucial peaks are highlighted in the spectra. (**D**) The thermal stability and the crystallinity of the conjugates was studied using differential scanning calorimetry (DSC) and the endotherm has been represented. (**E**) The secondary structural characteristics of Apo-bLf and Fe-bLf before and after Dox conjugation was studied using Circular Dichroism (CD) spectroscopy. CD values were obtained for secondary structure prediction at a wavelength range of 250 nm to 190 nm at an interval rate of 0.1 nm/s and the resultant CD spectra of milli degrees vs wavelength was plotted in the graph. The functional stability of Dox in the form of conjugates was confirmed by its ability to inhibit human topoisomerase II (TOPO II) to decatenated human kinetochore DNA (kDNA). The presence of nicked circular kDNA and relaxed circular kDNA was considered as a positive for the activity of TOPO II uninhibited by Dox. The presence of only the catenated kDNA at the wells is considered complete inhibition of TOPO II by Dox.

Dox was conjugated to Apo-bLf and Fe-bLf, using NHS-ester mediated conjugation, which resulted in the formation of two protein drug conjugates, Apo-bLf-Dox and Fe-bLf-Dox (Fig. 1). The SDS-PAGE (Fig. 1A) shows a single dark prominent band for bLf ~78 kDa for both pure bLf sample s as well as their conjugates. Further absence of any prominent higher bands in the 150 kDa region in SDS-PAGE confirms that there was no protein-protein coupling induced during the EDC-NHS coupling procedure. Western blotting using anti-bLf antibody (Fig. 1B) confirmed that the obtained conjugates contain pure bLf molecules in its active form.

### Physico-chemical characterisation confirms the chemical conjugation of Dox to bLf

Fourier Transform Infra-Red Spectroscopy (FTIR) confirmed the formation of amide bonds and a conjugation between bLf and Dox Fig. 1C. The presence of amide I and amide II vibrations in the spectra of both Apo-bLf-Dox and Fe-bLf-Dox at 1700 cm^−1^ and 1500 cm^−1^, which are the C=O stretch of free and quinone bound carboxyl group, indicates that the protein structure in the conjugates was intact, because these peaks are characteristics of an intact α helix structure^29^. The peaks seen at the 870 cm^−1^ and 805 cm^−1^ of the only Dox spectra are completely dampened in the case of conjugates because of the conjugation of NH_2_ groups in Dox reacting with COOH group of the proteins. The presence of the out of plane O-H vibration at 895 cm^−1^ (characteristic of Dox)^30^ is conserved in the conjugates, indicating that bLf-Dox consists of Dox moieties as well confirming the conjugation.

Differential Scanning Calorimetry (DSC) thermograms (Fig. 1D) of pure Dox indicates a sharp melting endotherm at 140 °C that is a characteristic of crystalline, insoluble Dox. In the case of drug conjugates, the sharp peak of Dox has disappeared and merged with the broad protein endotherms at 70 °C. Circular Dichroism CD spectroscopy (Fig. 1E), revealed that the spectra of both Apo-bLf and Apo-bLf-Dox were identical with a broad trench between 230 nm and 200 nm similar to Fe-bLf and Fe-bLf-Dox. The identical molar elipticity values for the proteins and the conjugates at the crucial wavelengths such as 193 nm, 196 nm and 207 nm indicates no significant conformational change in the protein folding. Quantitative secondary structural analysis revealed an increase in the percentage of α-Helices from 25.8% to 39% accompanied by a reduction in β-sheets to 9% from 19% between Apo-bLf and Apo-bLf-Dox whereas the secondary structural quantification remained similar between Fe-bLf and Fe-bLf-Dox apart from the reduction in α-Helices from 27.6% to 21.9%.

In order to verify if the bound Dox in conjugates is still functional, its topoisomerase inhibitory activity was assessed (Fig. 1F). Catenated kinetochore DNA - kDNA (Lane 3) which on incubation with TOPO II, gets decatenated and runs as two distinct bands in the gel representing nicked circular kDNA and relaxed circular kDNA (Lane 4). This reactive formulation in lane 4 (kDNA + TOPO II) was then incubated with different concentrations of Dox, Apo-bLf-Dox and Fe-bLf-Dox. Dox (800 nM) completely inhibited the decatenation activity of TOPO II (Lane 5) with an intact kDNA appearing on the wells without any decatenated products. This effect of Dox was seen to be reduced upon lowering its concentration to 200 nM and 100 nM (Lane 6 and 7). Apo-bLf-Dox and Fe-bLf-Dox also showed a similar inhibition pattern of TOPO II (Lane 8–Lane 13).

### bLf-Dox conjugates help in longer retention of Dox within cancer cells, resulting in increased cytotoxicity

**Figure 2:**
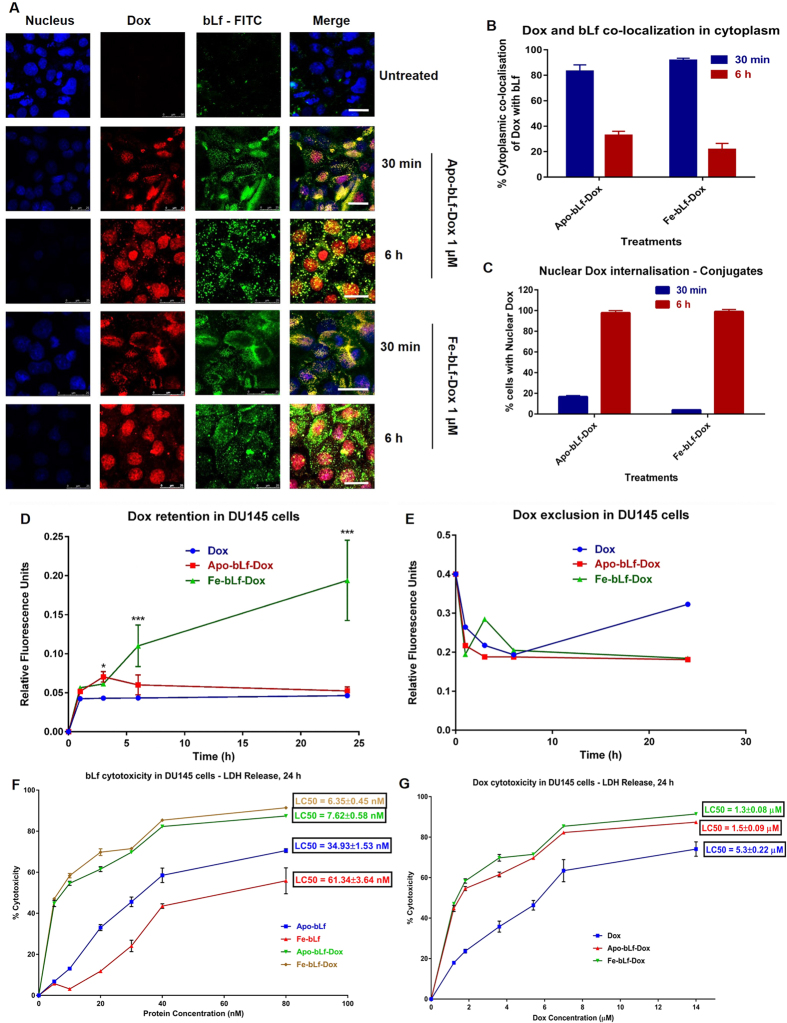
bLf-Dox conjugates promote greater retention of Dox resulting in enhanced cancer cytotoxicity. (**A**) Representative confocal microscopy images of DU145 cells showing the time dependent internalisation of Apo-bLf-Dox and Fe-bLf-Dox studied by immunofluorescence using goat anti-bLf primary antibody (1:100) and anti-goat IgG FITC conjugated secondary antibody (1:100). The internalisation of Dox was studied using its auto-fluorescence with excitation at 488 nm and emission at 630 nm. The nucleus was counterstained with DAPI indicated by the blue fluorescence along with a competitive binding to Dox. Scale = 25 μm. (**B**) The percentage of cells showing green and red fluorescence co-localised in cytoplasm (yellow) was considered as percentage bLf conjugated Dox plotted as histogram mean ± S.D. (**C**) Histogram showing presence of nuclear red fluorescence within the cells among 100 counted cells (mean ± S.D). (**D**) The amount of Dox present within the cells at various time points was considered as Dox retention and the relative fluorescence was plotted against time. Measurements were performed thrice in triplicates and the results are represented as mean ± S.D. (**E**) The amount of Dox present in the supernatant of the cells treated at various time points was considered as Dox retention and the relative fluorescence was plotted against time. Measurements were performed thrice in triplicates and the results are represented as mean ± S.D. (**F**) Cytotoxicity determination based on the LDH release from the cells post 24 h treatment at different concentrations in DU145 cells. The experiment was carried out thrice in triplicates. The X-axis is represented in terms of (**F**). bLf protein concentrations and (**G**). Dox concentrations. The determination of LC_50_ was carried out by non-linear regression fitted curve of the cytotoxicity against concentration and values are represented in the graph.

Cellular Dox content analysis revealed that Dox alone was taken quickly by cells within 30 min but it gradually tapered into a saturation phase over the 24 h period, thus giving an overall absence of cellular accumulation (Fig. 2D). The conjugates showed similar rate of accumulation within first 30 min without any significant difference, and cells showed an increased uptake at 3 h period (P < 0.05). Following this time point, accumulation of Dox using Apo-bLf-Dox conjugate tapered down until 24 h. However, Fe-bLf-Dox showed consistently improved accumulation of Dox within the cells until 24 h which was much higher (P < 0.001) than drug alone, at this time point. This phenomenon was also reflected in the Dox efflux assay which suggested that free Dox was easily excluded from the cells, in comparison to bLf-Dox conjugates (Fig. 2E).

In Fig. 2A confocal images show clearly the ability of the both Apo-bLf-Dox and Fe-bLf-Dox to internalise into the cells. A clear cytoplasmic co-localisation of bLf (green fluorescence, visualized for anti-bLf antibody) and inherent fluorescence of Dox (red), associated with its central anthracycline chromophore group was observed within first 30 min, indicating that bLf being analysed is indeed conjugated with Dox. At 6 h however, increased nuclear localisation of Dox was seen with a decreased co-localisation of Dox and bLf. During the first 30 min only 18.1% and 6.3% of cells showed nuclear Dox, which then increased to 98.2% and 99% after 6 h in both the treatments (Fig. 2B,C).

Figure 2F,G shows that there was a several fold increase in the cytotoxicity induced by both Dox as well as by bLf when used in the form of conjugates. LC_50_ value for Dox alone was 5.3 μM which decreased significantly to 1.5 μM and 1.3 μM in case of Apo-bLf-Dox and Fe-bLf-Dox, respectively. Importantly, Fe-bLf-Dox conjugates induced significantly lower cytotoxicity in non-cancerous RWPE-1 cells, in comparison to Dox alone as well as Apo-bLf-Dox treatments (Supplementary Figure 1).

### Increase in annexin-V expression and TUNEL^+^ cells confirms the induction of apoptosis

**Figure 3:**
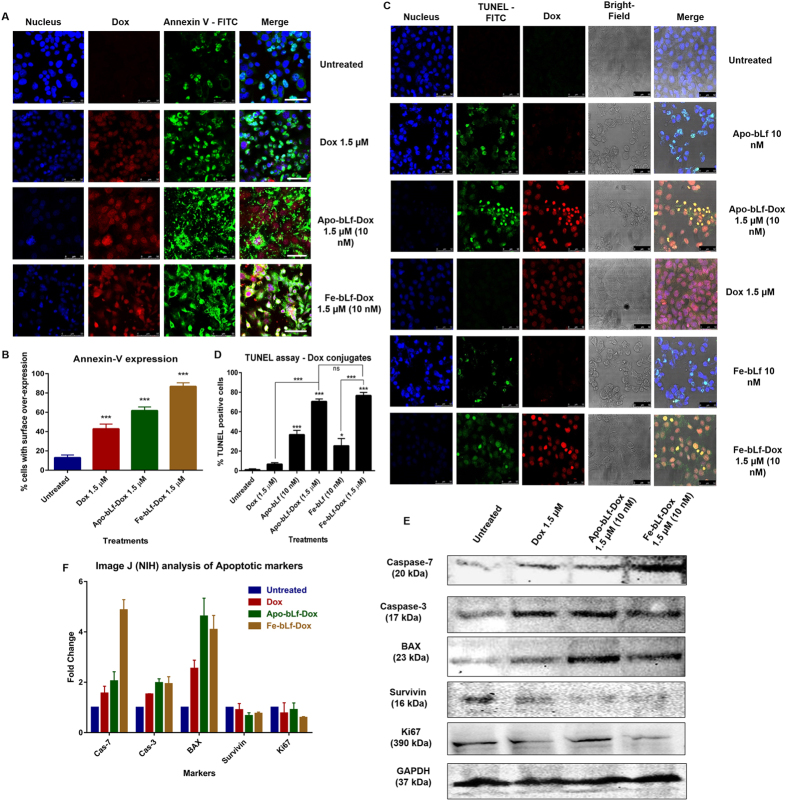
bLf-Dox conjugates treatment induces cancer cell apoptosis. (**A**) Representative confocal microscopy images of DU145 cells showing the expression of annexin-V as a marker of apoptosis post treatment with Dox, Apo-bLf-Dox and Fe-bLf-Dox. The annexin-V was stained using immunofluorescence with rabbit anti-annexin-V primary antibody and anti-rabbit-IgG FITC conjugated secondary antibody represented in green fluorescence. Dox auto-fluorescence is represented in red. The nucleus was counterstained with DAPI (blue). Scale bar = 50 μm. (**B**) Percentage of cellular annexin-V expression from 5 images each was calculated and represented as a histogram (Mean ± S.D.). (**C**) The presence of fragmented DNA as an end product of apoptotic cascade within the cell was considered as the confirmatory test for induction of apoptosis especially the Dox mediated DNA damage using TUNEL assay. The nucleus was counterstained with DAPI and Dox auto-fluorescence is represented in red. (**D**) Percentage of TUNEL positive cells from 5 images each has been represented as a histogram. Statistical analysis was performed using one-way ANOVA and a post-hoc Tukey’s test. (**E**) The molecular regulation of apoptosis was studied using Western blots for various apoptotic markers. The Western blotting was carried out with 100 μg of complete cell lysates against respective primary and secondary antibodies. (**F**) The Western blot images acquired were analysed for band density using ImageJ (NIH) software and the protein expression was given in relative fold change as compared to the untreated sample normalised against GAPDH and expressed as a histogram.

The representative confocal microscopy images (Fig. 3A) indicate that Apo-bLf-Dox and Fe-bLf-Dox (1.5 μM) induced higher apoptosis than Dox alone (increased number of cells showing higher green fluorescence intensity on cellular surfaces/membranes for annexin-V, represented as histogram in Fig. 3B. The percentage of TUNEL^+^ cells was significantly higher (P < 0.001) in Apo-bLf, Apo-bLf-Dox and Fe-bLf-Dox treatments than the Dox alone treatment (Fig. 3C,D).

Molecular analysis of apoptosis (Fig. 3E,F) showed an increase in caspase-7 expression upon all the treatments, with Fe-bLf-Dox showing maximum increase of about 5 fold. Nearly 2-fold increase in cleaved caspase-3 expression with both the conjugates treatments, suggests the activation of apoptosis cascade. An up-regulation of pro-apoptotic Bax expression in Apo-bLf-Dox and Fe-bLf-Dox (4.3 fold and 3.8 fold, respectively) treatments is much higher than 2.2-fold increase induced by Dox alone. Further survivin protein expression was highly down-regulated with Apo-bLf-Dox (0.61 fold) and Fe-bLf-Dox (0.66 fold), while there was a slight reduction with Dox treatment (0.92 fold). A similar trend was also observed in Ki67 expression which indicates negligible possibility of cell growth recovery, post bLf-Dox treatment induced apoptosis.

### bLf-Dox conjugates downregulate the drug resistance markers

**Figure 4:**
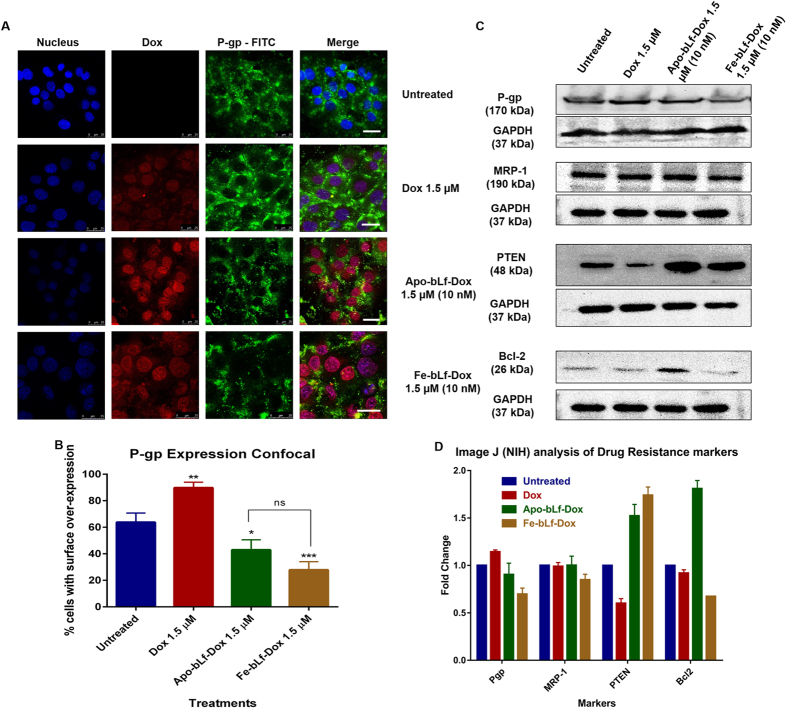
bLf-Dox conjugates are capable of overcoming molecular drug resistance pathway. (**A**) Representative confocal microscopy images of DU145 cells showing the expression of P-gp as a marker of drug resistance. The P-gp was stained using immunofluorescence with mouse anti-P-gp primary antibody and anti-mouse-IgG FITC secondary antibody visualised in green fluorescence. Dox–Red; Nucleus–Blue. Reduced DAPI fluorescence was observed in treatment involving Dox due to competitive nuclear binding. (**B**) Percentage of cellular P-gp expression was calculated as an average from 5 images for which data has been represented as a histogram (**B**). Statistical analysis was performed using multiple Student’s t-tests. (**C**) The molecular regulation of drug resistance was studied using Western blots for various drug resistance markers. The Western blotting was carried out with 100 μg of complete cell lysates against respective primary and secondary antibodies. (**D**) The Western blot images acquired were analysed for band density using Image J (NIH) software and the protein expression was given in relative fold change as compared to the untreated sample normalised against GAPDH and expressed as a histogram.

Dox being a substrate for the P-gp, increases cellular P-gp expression (Fig. 4A). Apo-bLf-Dox and Fe-bLf-Dox on the other hand did not increase the P-gp expression. On a closer analysis (Fig. 4B) it was also revealed that there was a significant reduction in the expression of P-gp, with bLf-Dox conjugates which were both much lower than P-gp expression in Dox treatments (P < 0.001) which showed a significant increase (P < 0.01) upon comparison with untreated cells.

Western blot showed a clear downregulation of P-gp expression to 0.86 and 0.69 fold for Apo-bLf-Dox and Fe-bLf-Dox, respectively as compared to untreated cells, which increased to 1.2 fold during the Dox alone treatment (Fig. 4C). The MRP-1 expression, also showed a decrease although only in the case of Fe-bLf-Dox to 0.8 fold.

The expression of PTEN tumour suppressor which decreased with Dox treatment (0.56 fold), while increased to 1.5 fold and 1.72 fold with Apo-bLf-Dox and Fe-bLf-Dox treatments, respectively, indicating that molecular regulation moving towards a reversal of drug resistance. As compared to untreated cells, Bcl-2 expression decreased with Fe-bLf-Dox (0.67 fold) and with Dox (0.91 fold).

### Advanced drug resistant (ADR1000-DU145) cells were sensitive to bLf-Dox treatments but not Dox alone

**Figure 5:**
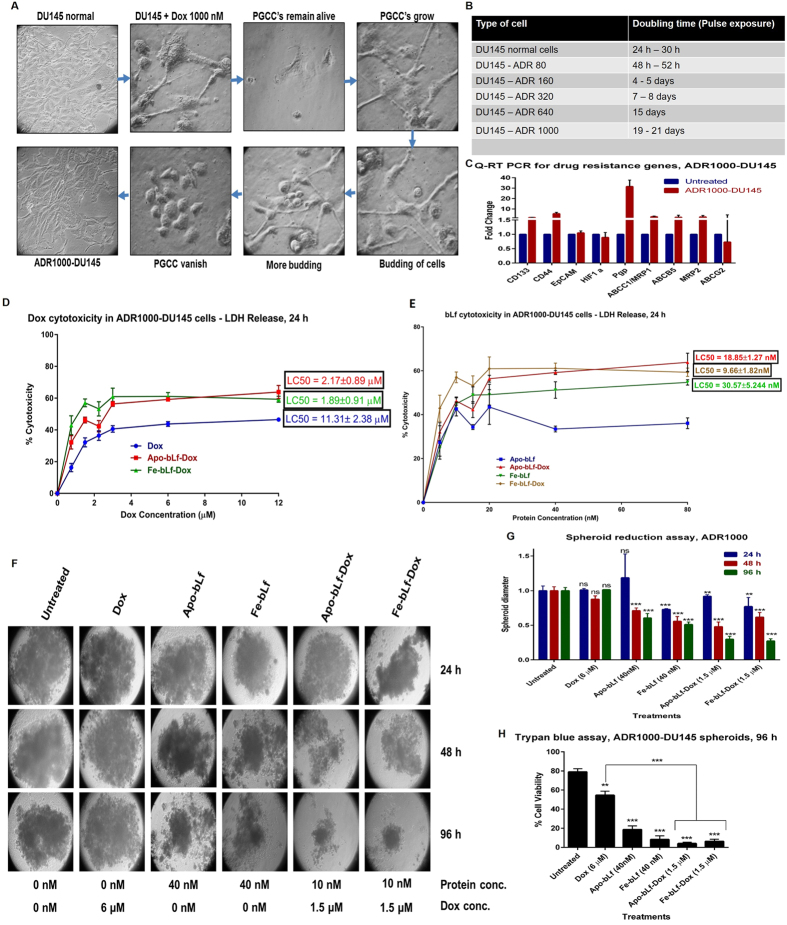
Advanced drug resistant (ADR1000-DU145) cells were sensitive to bLf-Dox treatments but not Dox alone. (**A**) The images represent the morphological changes in the DU145 cells upon subjected to pulse exposure of increasing concentration of Dox from 1 nM to 1000 nM. The cells accustomed to 640 nM Dox were treated with 1000 nM Dox on alternate days in complete growth media. The Dox treated DU145 cells were photographed every day following the first treatment and the different stages the cells undergo before they become accustomed to 1000 nM Dox has been represented. These cells were considered Advanced Dox Resistant at 1000 nM (ADR1000-DU145 cells). (**B**) Doubling time of cells subjected to development of chemo-resistance with Dox. (**C**) qRT-PCR was performed on the cDNA of DU145 cells and ADR 1000-DU145 cells. The relative fold expression changes of 9 genes of drug resistance and CSC markers were studied and is represented as bar graph (Livak Method). All values are represented as mean ± S.D and the experiment was done twice in duplicates. (**D**) Cytotoxicity of bLf-Dox conjugates on ADR1000-DU145 cells as a measure of LDH release from the cells was calculated after 24 h treatment at different concentrations. The LC_50_ values are represented mean ± S.D against respective Dox concentration. (**E**) Cytotoxicity (LC_50_) of bLf-Dox conjugates on ADR1000-DU145 cells represented as a function of protein concentrations. (**F**) ADR1000-DU145 cells were allowed to form prostaspheres for 7 days. Following spheroid formation, they were treated once at 0 h and again at 48 h and the spheroids were imaged under the microscope. (**G**) The size (Diameter) of the tumour spheroid was measured at 24 h, 48 h and 96 h post first treatment was measured using Image J (NIH) software tool. The experiment was carried out thrice with 5 spheroids per treatment. Two-way ANOVA with post-hoc Tukey’s test was used for statistical analysis. (**H**) Trypan blue dye exclusion analysis was performed post 96 h treatment to analyse the percentage viable cells left in the spheroids.

DU145 cells were grown under cytotoxic stress with pulse exposure of increasing Dox concentration upto 1000 nM and the surviving cells were drug resistant, ADR1000-DU145 cells. Figure 5B indicates, that with pulse exposure, doubling time of DU145 cells increased from 24 h for DU145 cells to 21 days for the ADR1000-DU145 cells. After few days of pulse exposure of 1000 nM Dox only, the polyploid giant cancer cells (PGCC) remained alive in culture which divided into a number of small cells forming the drug resistant subline ADR1000-DU145 (Fig. 5A). These cells displayed increased gene expressions of cancer stem cell markers CD44 and CD133 as well as several fold increase in the gene expressions of drug resistance genes, with P-gp expression especially showing 32-fold increase (Fig. 5C).

ADR1000-DU145 cells being an induced chemo-resistant cell line of DU145, showed very high LC_50_ against Dox equal to 11.31 μM, which was at-least 2 fold higher than LC_50_ of DU145 cells against Dox alone treatment (5.3 μM). This LC_50_ of ADR1000-DU145 cells against Dox reduced to 2.17 μM and 1.89 μM in Apo-bLf-Dox and Fe-bLf-Dox treatments, respectively (Fig. 5D,E).

Spheroid diameter analysis (Fig. 5F,G) shows that Dox at 6 μM concentration was least effective, with no significant reduction in spheroid diameter even after 96 h treatment. Apo-bLf-Dox and Fe-bLf-Dox were not significantly more effective than their corresponding protein alone treatments at 24 h and 48 h time-points whereas, after 96 h treatment both Apo-bLf-Dox and Fe-bLf-Dox showed significant spheroid size reduction compared to Apo-bLf and Fe-bLf, respectively (P < 0.05) as well as Dox (P < 0.001).

Further, effectiveness of bLf-Dox conjugates against drug resistant CD44+/EpCAM+ double positive cancer stem cell enriched DU145 cells was tested, which showed that both Apo-bLf-Dox and Fe-bLf-Dox were capable of inhibiting clonogenic, migration as well as 3D growth properties of these cells (Supplementary Figure 2).

### TRAMP mice showed prolonged survival and reduced tumour growth when treated with Fe-bLf-Dox conjugates

**Figure 6:**
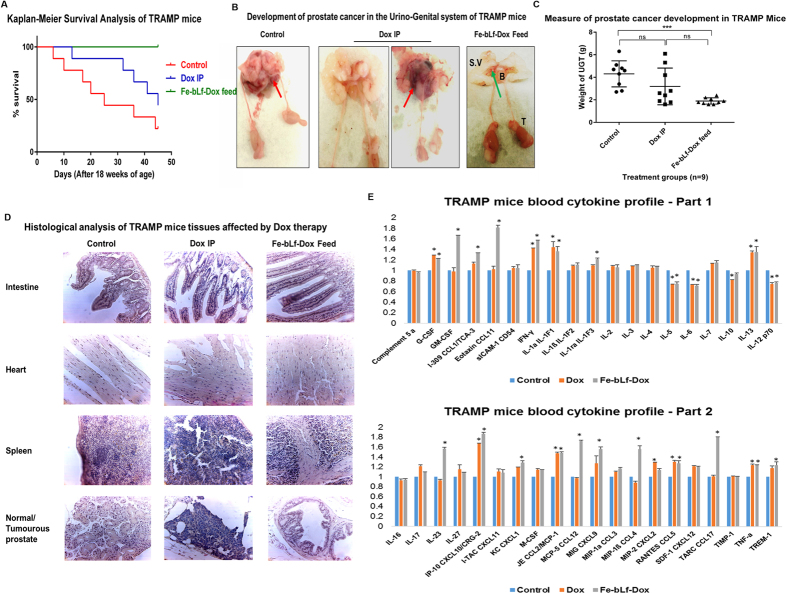
Fe-bLf-Dox conjugates inhibited tumour development and prolonged the survival of TRAMP mice. (**A**) Kaplan—Meier survival analysis of TRMP mice groups (Control, Dox IP and Fe-bLf-Dox diets) for n = 9 mice per group following treatment post 18 weeks of age for 45 days. (**B**) Dissected Urino-Genital Tract (UGT) of the TRAMP mice displaying seminal vesicles (S.V), bladder (B), testes (T) and the prostate (Red = Tumourous; Green = Normal). (**C**) Normalised UGT weights against the mice body weights as a measure of tumour development in TRAMP mice in control, Dox IP and Fe-bLf-Dox feed groups (n = 9). Two-way ANOVA was performed to evaluate statistical significance followed by post-hoc analysis by Tukey’s test. (**D**) Histopathological analysis of TRAMP mice tissues post haematoxylin and eosin staining viewed under optica light microscope 400X magnifications. Sections of mice intestine, heart, spleen and prostate are provided. (**E**) Proteome profiler array analysis of serum cytokine expression profile of TRAMP mice treated with Dox and Fe-bLf-Dox (n = 3). Image J analysis of micro-array profile was carried out and relative fold change in the integrated density of each cytokine has been represented as histogram (Mean ± S.D.).

Transgenic Mice exhibiting Adenocarcinoma of Prostate (TRAMP mice), were employed further to study therapeutic effectiveness of Fe-bLf-Dox upon oral feeding. Only Fe-bLf-Dox was chosen for animal study due to its lower toxicity exhibited on normal RWPE-1 cells compared to Apo-bLf-Dox (Figure S-1). These mice are capable of developing prostate cancer spontaneously after puberty^31^. Treatments were started at the age of 18 weeks and continued till 24^th^ week, beyond which prostatic adenoma formed is expected to metastasize. All TRAMP mice treated with Fe-bLf-Dox (n = 9) survived until to 24 weeks of age, in comparison to 3/9 mice that survived in control group and 5/9 mice in Dox IP treated group (Fig. 6A). Control group mice developed large tumours (Fig. 6B) due to which they were humanely killed during study period. Although only 3/9 Dox IP treated mice developed large tumours as seen in the measure of Urino-Genital Tract (UGT) weights (Fig. 6C), the percentage survival was lower in this mice group due to significant body weight loss and hair loss observed in mice of this group. Fe-bLf-Dox treated mice had significantly reduced the UGT weight than control mice (P < 0.001) thereby suggesting that Fe-bLf-Dox was more effective in inhibiting tumour development than Dox alone injections, which showed no significant reduction in tumour weight in comparison to control (Fig. 6C).

### Fe-bLf-Dox fed mice showed normal histology and blood composition, with improved anti-tumour cytokine response, whereas Dox alone showed several side effects

Apart from the general observation of large tumour appearing in the prostate gland of control mice, other abnormalities were observed in the Dox IP treated animals. Most importantly observed differences were the enlarged heart and spleen in Dox IP treatments, relative to control or Fe-bLf-Dox treated mice. Although there was no significant difference in the heart weight between the treatments, histopathological analysis of the heart displayed a larger and irregular cell structure with Dox IP treatment. On the other hand, regular aligned cardiomyocyte arrangement was observed in the Fe-bLf-Dox treated mice in relation to the control mice. Similarly, larger spleens were found in the Dox IP treated mice. The histological analysis shows splenic damage after Dox IP treatment whereas Fe-bLf-Dox treatment showed normal spleen pathology as of the control group (Fig. 6D). Histology of the intestines revealed no significant destruction to the intestinal villi after Fe-bLf-Dox feeding. The prostate histology sections also revealed a higher grade tumorous architecture in the control and Dox IP treated groups, in comparison to the Fe-bLf-Dox fed mice (Fig. 6D). Apart from these anomalies, the total blood analysis revealed that Dox IP treated mice had abnormally low RBC count, haemoglobin content and haematocrit content which were at normal levels for Fe-bLf-Dox treated mice (Table 1).

Upon analysing the cytokine mediated immune response of TRAMP mice mediated by treatments (Fig. 6E), it was clearly visible that Fe-bLf-Dox was capable of triggering greater anti-tumour immune response in mice than Dox treatment. Both Dox and Fe-bLf- Dox significantly increased the protein expression of G-CSF, IFN-γ, IL-1a, IL-13, IP-10, CCL2, CCL5 and TNF-α (P < 0.05), whereas both treatments reduced the expression of IL-5, IL-6 and IL-12. More importantly, Fe-bLf Dox was capable of inducing greater expression of anti-tumour and macrophage homing chemokines such as GM-CSF (1.7 fold), CCL11 (1.8 fold), IL-1ra (1.3 fold), IL-23 (1.5 fold), CCL12 (1.8 fold), CCL4 (1.5 fold) and CCL17 (1.7 fold), than Dox injections.

## Discussion

Multi-drug resistance (MDR) is the major issue, apart from the unpleasant and detrimental side effects concerning the long term use of cancer chemotherapeutics. Hence there is a focus on novel therapeutics development that can provide effective therapy even in case of drug resistant cancer phenotypes. Dox is a drug, notorious for its ability to induce chemo-resistance in tumours along with cardiotoxicity and requires effective strategies to overcome it. Several drug conjugates have been developed by researchers to enhance their functionality, and our laboratory has also shown that siRNA-EpCAM aptamer conjugates^32^ and various chimeric aptamers can be the next novel class effective therapeutics^33^.

Cellular internalisation of bLf has been highly characterised and found to be facilitated by receptor mediated endocytosis^17^,^28^. As a complex of protein-drug conjugate, Dox can enter the cells through receptor mediated endocytosis facilitated by Apo-bLf and Fe-bLf undetected by the membrane transporter pumps/proteins. This can also facilitate a longer retention of drug within the cells^34^. bLf was therefore employed in novel drug conjugation strategy to improve Dox cytotoxicity against prostate cancer.

Bovine lactoferrin in its iron free (Apo-bLf) and iron saturated (Fe-bLf) forms, was conjugated to Dox using EDC-NHS mediated cleavable amide bond formation in an attempt to exploit the anti-drug resistance and receptor mediated cellular internalisation properties of these bLf forms in delivering Dox. There was no degradation of the proteins during the conjugation process suggested by the absence of any smaller bands in the bLf-Dox in SDS-PAGE.

FTIR spectra of conjugates confirmed the presence of stably conjugated Dox by Dox specific out of plane OH stretching at 895 cm^−1 ^30, which was otherwise absent in protein only samples. Since it is important for a drug to be amorphous in order to have greater solubility to reach the targets through the blood stream^35^, DSC thermograms indicate a shift from crystalline to amorphous form of Dox post conjugation, resulting in increased solubility of the conjugated drug observed through the peak broadening^36^. CD spectra suggested preservation of the secondary structure of both Apo-bLf and Fe-bLf post conjugation with Dox. The increase in percentage α-Helices in Apo-bLf-Dox is due to the stabilizing effect of Dox binding on the structure of Apo-bLf, which otherwise has a an open unfolded structure as against Native bLf. However, the changes in the secondary structural composition of Fe-bLf-Dox was not significant. Due to the similarity in structures of bLf and bLf-Dox, (irrespective of it being in iron free or in iron saturated form) CD studies indicate that secondary structure of bLf is mostly not affected by the process of conjugation with Dox. At 800 nM concentration, the ability of Dox to inhibit the decatenation activity of human DNA topoisomerase II on catenated kDNA, suggests that the Dox present in the conjugates is in its active form behaving in the same manner as that of free Dox^16^. Thus bLf-Dox synthesised was pure, non-degraded and active.

The analysis of intracellular retention of Dox within DU145 indicated that improved intracellular retention of Dox (significantly by several fold), when delivered as conjugates especially with Fe-bLf-Dox. Fe-bLf uptake is characterised by gradual constant, receptor mediate uptake resulting in an increased retention up to 24 h. In a complementary study, ability of DU145 cells to pump Dox out of cells was analysed with Dox alone treatments releasing maximum Dox back into cell media during the 24 h period, indicating the activity of membrane efflux pumps in removing cellular Dox which was suppressed in the case of bLf-Dox conjugates. On a bigger picture however, Fe-bLf-Dox showed greater retention in DU145 than its iron-free counterpart because Fe-bLf preferentially used the Lf receptors whereas Apo-bLf could be internalised through other receptors (unpublished observations).

Following receptor mediated endocytosis, bLf enters the endo-lysosome^21^ which could cause the cleavage of the drug from the protein due to the action of the digestive enzymes and the acidic environment leading to hydrolysis of covalent bond^37^. This results in nuclear Dox accumulation which was confirmed by reduced fluorescence of nuclear counterstain DAPI due to the competitive binding by Dox, proving our hypothesis that bLf-Dox leads to the increased retention of the drug. Thus bLf-Dox conjugates can also be considered as pro-drugs for Dox however; in this case bLf is also a functional component unlike in other pro-drugs.

Due to their enhanced cellular uptake leading to reduced drug efflux, and further combined with cytotoxic nature of both bLf and Dox, the reduction in LC_50_ of Dox by more than 4 fold was very prominent, similar to the reduction in the LC_50_ value of Dox when co-treated with P-gp inhibitor such as carvedilol or verapamil^38^. This implied a role played by bLf as a P-gp suppressor. There is also another possibility that the conjugates by-pass the P-gp activity because protein sequesters Dox from acting as a substrate for P-gp. However, the fact that there was no Dox efflux even after 24 h, suggests that P-gp bypass could not be the only mechanism, because if there was no down-regulation of P-gp, then after 6 h, free Dox would still be available for P-gp to pump it out of the cells.

The above observations lead to the conclusion that both the down-regulation of P-gp as well as the bypass of P-gp takes place with bLf-Dox confirmed by the immunofluorescence images which indicate that P-gp expression increases upon exposure to Dox alone. This is because Dox is a prominent substrate of P-gp causing its transcription as soon as Dox gets detected in the tumour cell and its microenvironment^39^. This does not happen in the case of the conjugates which prove the point that bLf-Dox conjugates can by-pass the P-gp drug efflux mechanism. However, it can also be noted that the expression of P-gp in cells treated with Apo-bLf-Dox and Fe-bLf-Dox was further lower than the untreated cells, indicating that these bLf-Dox conjugates are also capable of down-regulating the P-gp expression. This results in the observed preferential retention of the bLf-Dox conjugates within the cell causing greater cancer cell death. The P-gp targeting activity of bLf was similar to that of the Akt inhibitor Perifosine that can down-regulate P-gp expression indirectly and induce apoptosis by an indirect independent mechanism^40^.

**Figure 7:**
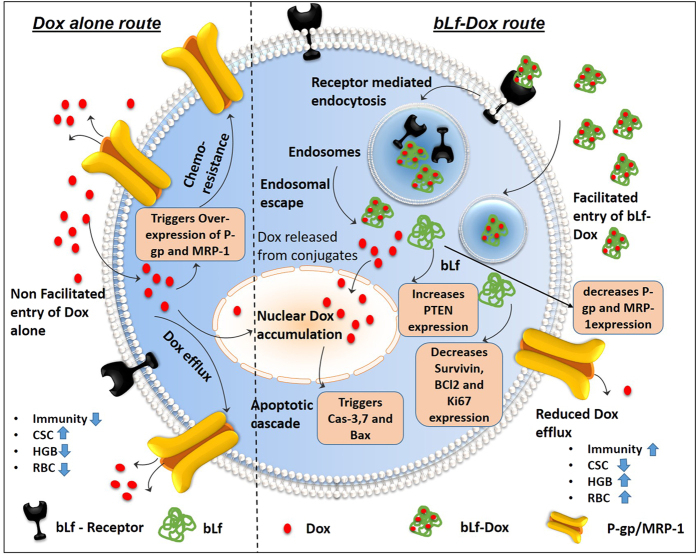
Schematic representation summarizing the inferences of this study about the role of Fe-bLf-Dox conjugates in overcoming drug resistance. Dox alone (Left) causes increase in chemo-resistance by up-regulating P-gp and MRP-1 expression thereby getting effluxed out of the cancer cells quickly causing minimal damage. bLf-Dox conjugates are easily internalised by cancer cells without triggering P-gp increase and facilitates longer nuclear retention of Dox increasing its cytotoxicity. Meanwhile bLf also decreases survivin, P-gp and MRP-1 expression and increases PTEN expression to decrease chemo-resistance and trigger cancer cell apoptosis. Fe-bLf-Dox treatment in TRAMP mice also reversed the RBC and HGB loss due to Dox treatments and induced anti-tumour immunogenicity. The schematic illustration was generated by modifying images purchased in the PPT Drawing Toolkits-BIOLOGY Bundle from Motifolio, Inc.

The loss of PTEN function due to the Bcl-2 overexpression is the main cause for the elevation of drug resistance in cancer^41^. The over-expression of PTEN in turn supresses Bcl-2 mediated anti-apoptotic activity in these cells where p53 is constitutively mutated^42^. Bcl-2 and MRP-1 are also a major proteins contributing to the Dox and cisplatin induced chemo-resistance and a replacement of Bcl-2 was able to restore the chemo-sensitivity to Dox in chondrosarcoma^43^. Fe-bLf-Dox was seen to be most effective in reducing the Bcl-2 expression levels accompanied by the increase in PTEN expression without any increase to MRP-1 levels, and a slight decrease in MRP-1 expression with Fe-bLF-Dox to 0.8 fold was observed. Considering the fact that the bLf-Dox conjugates increase the retention of Dox in the cells, it is natural that the cellular response would be to increase MRP-1 to several fold which hasn’t happened in this case. While, the released Dox from the conjugates continues to elevate the MRP-1 levels, the bLf component appears to regulate its expression. Fe-bLf on its own is capable of reducing MRP-1 expression in DU145 cells (unpublished observations from our lab). This implied that bLf-Dox conjugates can overcome drug resistance mechanism by down-regulating P-gp and MRP-1 via inhibiting the mTOR pathway described schematically in Fig. 7.

The induction of apoptosis was confirmed using TUNEL assay. DNA fragmentation is a characteristic of Dox induced cell apoptosis due to topoisomerase inhibition^44^ and was found to be induced at a much higher frequency in case of conjugates in contrast to Dox alone. Dox, apart from being a topoisomerase inhibitor, also induces caspase dependent apoptosis that can be reversed by the over-expression of anti-apoptotic molecules leading to cellular senescence^45^. We also noted that the ability of bLf to reduce survivin expression has been retained even in the form of Dox conjugates, suggesting that the functional aspect of the bLf as an anti-survivin bio-molecule is retained.

As a whole in this study, Fe-bLf-Dox was seen to be more potent than Apo-bLf-Dox. Iron also plays a major role in this enhanced toxicity displayed by Fe-bLf, because iron is a crucial effector of Dox induced cytotoxicity^46^. Further, during Dox chemotherapy there is a severe loss of serum iron, haemoglobin and RBC content which can be overcome by Fe-bLf-Dox. Tumour cells, due to their requirement of high iron content, display ability to uptake enormous quantity of iron complexes via transferrin receptors^47^. Hence DU145 cells internalise and retain the iron containing Fe-bLf-Dox for a longer period and to a greater extent thereby further increasing the potential of Fe-bLf-Dox within the tumour. This confirmed the study of role played by iron in enhancing the ability of bLf in functioning as an adjuvant for chemotherapy. Iron saturation in bLf enhanced its chemosensitisation properties in lymphomas and B16 melanomas thereby, Fe-bLf proving to be more effective than Apo-bLf^27^.

The results obtained using CD44+/EpCAM+ double positive DU145 cancer stem cells concurred with the fact that bLf-Dox conjugates were more effective than Dox alone in reducing the aggressiveness of resistant cancer stem like cells by inhibiting their migration and tumour formation (Supplementary Information). In an attempt to generate a drug resistant phenotype of DU145 cells, the clinically relevant pulse exposure method was attempted for developing (advanced Dox resistant) ADR1000-DU145 cells^48^. The change in the morphology of the DU145 cells post-exposure to increasing concentration of Dox was an indication of cytotoxic stress related cellular adaptation. The presence of PGCCs (Polyploid Giant Cancer Cells) is a characteristic response of cellular survival post hypoxic or cytotoxic stress^49^. PGCCs then undergo budding and form many small daughter cells which then develop into mono nuclear resistant cells that were present in ADR1000-DU145 cells.

The 2D cell cytotoxicity assay using ADR1000-DU145 revealed a higher resistance shown by these cells against Dox treatment, with an increase in the LC_50_ value for Dox to be increased by at-least 2 fold to 11.31 μM. Evidence shows that laboratory generated drug resistance cancer cell phenotypes showed about 5 to 10 fold increase in their LC_50_ value^50^ and a similar adriamycin resistant phenotype of DU145 cells showed 263 fold increase in LC_50_ value for Dox to 42 nM^48^. The resultant LC_50_ in current study was lowered when bLf-Dox conjugates were used, that indicated that ability of bLf to reduce P-gp and MRP-1 expression thereby sufficient to restore the sensitivity of ADR1000-DU145 cells towards Dox. Similar results were also obtained in the 3D spheroid assay, thus displaying success in solid tumours.

The *in vitro* experimental findings were further augmented by observations in TRAMP mice models which showed a complete inhibition of tumour development in Fe-bLf-Dox treated group, at the same total concentration of Dox alone in IP injection. Dox in general is very toxic to normal cells especially in cell culture since it targets cell proliferation^51^. Since Fe-bLf-Dox conjugate induced significantly lower toxicities in normal RWPE-1 cells, compared to either Dox alone or Apo-bLf-Dox and hence Fe-bLf-Dox was only considered for mice study. Generally, around 24 weeks of age Male TRAMP mice harbour large solid prostate tumours, which were completely absent in the Fe-bLf-Dox conjugates. More importantly, the reduced side effects of chemotherapy upon treatment with cytotoxic drugs observed with the use of Fe-bLf-Dox prompts to a target specific enhanced functionality of the bLf-Dox conjugates.

Analysis of serum cytokine profiles revealed that Fe-bLf-Dox was capable of triggering an anti-cancer immune response as against that of Dox alone injections owing to the immuno-modulatory activity of Fe-bLf^17^. The lack of complement activation seen from the low complement 5a value indicates a lack of non-specific inflammatory response being induced apart from indicating that purified Fe-bLf-Dox was free of endotoxin contamination. Pro-inflammatory cytokines such as IL-5, IL-6 and IL-17 are capable of promoting tumour growth which were reduced significantly by Fe-bLf-Dox. The serum levels of tumour inhibiting cytokines such as IFN-γ and TNF-α^52^ were elevated significantly by both Dox as well as Fe-bLf-Dox compared to control group. However, between the Dox and Fe-bLf-Dox treatments no significant difference in TNF-α levels was observed while IFN-γ levels elevated significantly (p < 0.05) with Fe-bLf-Dox. bLf being an innate immunity component is widely studied as an immumodulatory protein^17^. However, there are no conclusive reports on the TNF-α response upon bLf exposure. Also, the research data about effect of Fe-bLf on cytokine and chemokine levels in anti-tumour immunity is scarce. We have shown earlier that Fe-bLf can induce TNF-α production as an anti-tumour response but it was not much different from that associated with Dox induced increase^27^. Hence TNF-α expression seen in this study is likely a sum total of Dox as well as Fe-bLf triggered levels. Yet, similar to that of TNF-α, deeper analysis is required to understand why the reason for such subtle changes. The cytokine analysis data in this study shows that there was not any adverse inflammatory response in mice upon the introduction of Fe-bLf to Dox while a favourable response towards the generation of anti-tumour cytokine profile was seen. Future detailed investigations are warranted to analyse the immune-regulatory property of Fe-bLf-dox conjugates so that contribution of each component to the generation of cytokine response can provide a clear picture of why there is no change in the TNF-α levels between Dox and Fe-bLf-Dox treatment.

Chemokines that work in the favour of recruiting tumour homing cytotoxic T-lymphocytes and macrophages such as GM-CSF, CCL17 and CCL4 were increased specifically in Fe-bLf-Dox treatment. On the other hand, significantly increased expression levels of CCL11 and CCL12 which are sometimes associated with cancer invasiveness^53^ were also seen during Fe-bLf-Dox treatment, which could be a survival response from the stressed tumours. Since CCL11 is known for its neurodegenerative and allergic inflammation activities, paraffin embedded mice brain sections were stained with anti-mouse Mouse CCL11/Eotaxin antibody. Interestingly, in contrast to high serum levels, Fe-bLf-Dox conjugate treated mice brain tissue revealed no CCL11 expression, no signs of neurotoxicity while low to moderate CCL11 expression in Dox treated mice brain tissue was seen (with evident neuronal loss), in comparison to a very low CCL11 expression in control mice brain (Supplementary Figure 3). As discussed in the Supplementary information, the retained neuroprotective ability of Fe-bLf, while fed orally as a dug conjugate to circumvent Dox induced neurotoxicity, is an interesting finding that warrants more research. Hence, further follow up investigations of Fe-bLf and Fe-bLf-Dox may prove the conjugate as very effective multi-functional pro-drug for the treatment of prostate cancer.

### Conclusion and Future Perspectives

A successful synthesis of a bLf-Dox conjugate resulted in a pure and functional bio-conjugate of bLf and the drug doxorubicin (Dox). Both Apo-bLf-Dox and Fe-bLf-Dox induced increased cytotoxicity in DU145 prostate cancer cells when compared to Dox alone. However, on a molecular basis the Fe-bLf-Dox induced significant apoptosis and increased chemo-sensitivity than Apo-bLf-Dox in DU145 cells, apart from being effective against the drug resistant ADR1000-DU145 cells. Taking into consideration the lower toxicity of Fe-bLf-Dox in non-cancerous RWPE-1 cell line than Dox and Apo-bLf-Dox, we conclude that Fe-bLf-Dox can be a vital cog as an improved future chemotherapeutic against prostate cancer. Targeted oral nano-formulations can in future be used to deliver the promising drug payloads towards prostate cancer. Further considering the improvement in RBC levels and the cytokine response in mice, induced by the Fe-bLf Dox treatments, the next ideal step would be to take it to the clinical trials for patients undergoing chemotherapy.

## Methods

### Preparation of bLf-Dox conjugates

Doxorubicin (Dox) and bovine lactoferrin (bLf) conjugates were prepared to improve the retention of Dox within cancer cells and to improve its therapeutic efficiency. 5 mL of 80 mg/mL bLf solution Apo-bLf and Fe-bLf) was taken. Apo-bLf and Fe-bLf were prepared from commercially available health grade native bLf (Australia’s Own Pty. Ltd) according to the protocol^27^. To these protein solutions, around 0.4 mg of 1-Ethyl-3-(3-dimethylaminopropyl) carbodiimide (EDC) (Sigma-Aldrich, Australia)/mg of protein was added and 0.6 mg of N-Hydroxysuccinimide (NHS) (Sigma-Aldrich, Australia) per mg of protein was supplemented. The mixture was allowed to stir at room temperature for 1 h and pH was maintained at 8.0. After 1 h, reaction was arrested using 10 μL of 2 mercaptoethanol (Sigma-Aldrich, Australia). To the mixture, 4 mg of pure Dox (Sigma-Aldrich, Australia) dissolved in 400 μL of DMSO was added and mixture was further incubated with constant stirring at 17 °C. Excess reagents were removed using dialysis against water at 4 °C for 48 h using a 10 kDa molecular weight cut-off dialysis membrane (Spectrum Labs, Australia). The conjugates were then freeze dried and subjected to further characterisation.

### DNA decatenation assay to study Dox activity

In order to determine if the doxorubicin is still active, bLf-Dox conjugates were tested for their topoisomerase inhibition activity i*n vitro* using human topoisomerase assay kit (Topogen, USA)^54^. Briefly, Human Topoisomerase II Enzyme was incubated with 0.2 μg of kinetoplast DNA (kDNA) for 30 min at 37 °C using assay buffer supplied with the kit. In case of treatments, the mixture of kDNA and topoisomerase was also incubated with varying concentrations of Dox in the form of drug alone or in the form of the conjugates. The enzyme concentration was maintained at 0.2 units per μL. Reactions were terminated using stop buffer and loaded directly onto a 1% agarose gel containing 1X SYBR safe dye. After electrophoresis, the gel was photographed using Chemi-doc XRS+ gel documentation system.

### Fourier Transform Infra-Red spectroscopy (FTIR)

Apo-bLf, Fe-bLf, Apo-bLf-Dox and Fe-bLf-Dox and Dox samples were mixed individually with 200 mg of KBr powder (Sigma-Aldrich, Australia) and pelleted into a KBr disc using a hydraulic press^55^,^56^. FTIR spectroscopy (Bio-Rad with OPUS 5.5 software) analysis was performed between 4000 and 450 cm^−1^ at a resolution of 4 cm^−1^ averaging 10 scans.

### Differential Scanning Calorimetry

5 mg of each sample was measured accurately by sensitive balance and sealed into an aluminium pan. DSC (TA instrument DSC Q200) scans were programmed in the temperature range of −50−175 °C and at heating rate of 10 °C min^−1^. Plain sealed aluminium pan was kept as the reference for measuring the heat flow. A graph of heat flow vs temperature was plotted for analysis.

### Maintenance and subculturing of cells

DU145 cells were obtained from ATCC (HTB–81) supplied by Cryosite, Australia. RWPE-1 cells were provided as a kind gift by Prof. Gail P. Risbridger’s laboratory of Monash University, Australia. DU145 cells were grown in minimum essential media with Earle’s salts (EMEM) containing 10% foetal bovine serum (FBS) and 1% antibiotic (Penicillin-Streptomycin). RWPE-1 cells were grown in keratinocyte serum free medium (KSFM) containing the supplements 0.05 mg/mL bovine pituitary extract (BPE) and 5 ng/mL epidermal growth factor (EGF). The cells were grown at standard conditions of 37 °C and 5% CO_2_ in a humidified cell culture incubator (Thermoline, Australia). All the reagents for cell culture were tissue culture grade and purchased from, Invitrogen, Life Technologies, Australia.

### Development of advanced drug resistance DU145 cells - ADR1000-DU145 cells

In order to mimic the formation of drug resistant prostate cancer cells, DU145 cells were forced to grow under constant cytotoxic stress to increase their resistance against cytotoxic drugs. The pulse exposure method is the clinically most relevant method for inducing drug resistance^57^. In this method, DU145 cells were incubated with increasing concentration of Dox (80 nM, 160 nM, 320 nM, 640 nM, 1000 nM) on alternate passages with time given for the cells to recover from cytotoxic shock for a day. The cell lineage at the end of the 1000 nM treatment were considered to be advanced drug resistant to Dox (ADR1000-DU145). These cells were then continuously cultured by the pulse exposure method at 1000 nM of Dox.

### Internalisation of bLf and bLf-Dox in DU145 cells

Cellular uptake of Apo-bLf, Fe-bLf and Apo-bLf-Dox and Fe-bLf-Dox was studied by immunofluorescence and visualized by confocal microscopy^56^. DU145 cells were seeded in 8- -well slides at a density of 1 × 10^5^ cells/well and were allowed to grow for 1 day. They were then treated with 10 nM of the different bLf forms for different time intervals in complete growth media. Following treatment, medium was removed, and cells were washed thoroughly using PBS (pH 7.4) to remove unbound and non-internalized bLf from the cell layer, followed by fixation with 4% paraformaldehyde. The cells were permeabilised with 0.1% TritonX-100 for 5 min on ice. Cells were then incubated with primary antibody, goat anti-bovine lactoferrin at a dilution of 1:200 in PBS at 37 °C for 1 h. The primary antibody was then removed and after washing, cells were incubated with anti-goat IgG-FITC (1:100) conjugate and counterstained for nucleus with DAPI in fluorshield. Untreated cells and cells without primary antibody were used as control. The slides were imaged using TCS SP5 Leica broadband confocal microscope and processed using LAS-AF software. Dox was visualised using its autofluorescence with excitation at 488 nm and emission between 570–630 nm. Other immunofluorescence based assays were also performed using the same procedure with respective primary and secondary antibodies.

### Analysis of cellular Dox content and Dox exclusion

DU145 cells were plated out in a 96 well plate at a density of 1 × 10^4^ cells per well and allowed to grow overnight. On the following day, the cells were treated with 1 μM concentration of Dox both in the form of drug alone as well as in the form of conjugates for different time points of 30 min, 1 h, 3 h, 6 h, 12 h and 24 h. Following the treatments, media containing treatments were removed and replaced with fresh media for 2 h. Finally, all media were aspirated out of the wells and the cells were permeabilised using 0.1% triton-X-100 for 30 min at 37 °C. After the permeabilisation, amount of Dox present inside cells was measured using fluorescence with excitation at 480 nm and emission at 630 nm. The amount of Dox present in the supernatant was also measured as excluded Dox by the cells.

### Tumour spheroid/Prostasphere culture

The DU145/ADR1000-DU145 cells were trypsinized using the routine protocol and 500 cells/well were plated in 1% agarose-coated 96 well plates for adherence free growth^58^. The coated agarose plates were sterilised under UV for 1 h before plating out cells which were then allowed to form prostaspheres for 7 days under constant monitoring with light microscope every day. The media was changed every 3^rd^ day to allow healthy spheroid formation from the cells. After 7 days, the tumour spheroids were treated with varying concentrations of desired treatments. After incubation with treatments, the tumour spheroids were imaged by light microscopy and the diameter of the spheroids was calculated as a measure of tumour growth using ImageJ (National Institute of Health, USA). The values were then plotted as a histogram.

### *In vivo* experimentation on TRAMP mice

All animal experiments were performed within the guidelines of Australian Code for the Care and Use of Animals for Scientific Purposes (8^th^ Ed) and by following the 3R guidelines. The experiments were approved by Animal Ethics Committee, Geelong (AECG) of Deakin University, Australia under the ethics approval number AEC G28/2014. Wild type C57BL/6j mice were obtained from Animal Research Centre (ARC, Perth) and TRAMP mice were obtained from Dr. Michael Cater of Deakin University. For breeding purposes, one pair of animals (Male WT-C57BL/6j and Female homozygous TRAMP) was housed in a cage for 6 weeks for breeding. The heterozygous C57BL/6j x TRAMP mice offspring of 18 weeks’ age (n = 9) were used for the study. After 18 weeks, control group mice were fed normal diet, whereas mice in Dox alone group were injected with a single I.P injection of 15 mg/kg Doxorubicin. The Fe-bLf-Dox was prepared as an oral formulation in AIN93G basic diet and fed to the animals at a dose of 15 mg/kg. The survival and health of animals were monitored daily until the age of 24 weeks beyond which they were all humanely killed and analysed. The urino-genital tract (UGT) was extracted from each animal and was weighed as a measure of tumour development normalised against the bodyweight of mice^59^. Histology sections obtained for all vital organs were analysed. Blood was taken by terminal cardiac puncture, serum was isolated and final blood & serum analysis was carried out.

### Statistical Analysis

All data obtained are expressed as mean ± S.D. One-way ANOVA/Two-way ANOVA was used depending on the number of variable parameters, followed by post-Hoc Tukey’s test. Values of *p ≤ 0.05, **0.05 < P < 0.01 and ***0.01 < P < 0.001 were considered significant. Statistical analyses were carried out using GraphPad Prism 6.0 software. The analysis of cytotoxicity (LC_50_) was performed using a four-parameter sigmoidal non-linear regression based calculation of LD_50/_LC_50_ using Graphpad prism software.

## Additional Information

**How to cite this article**: Shankaranarayanan, J. S. *et al*. Doxorubicin Conjugated to Immunomodulatory Anticancer Lactoferrin Displays Improved Cytotoxicity Overcoming Prostate Cancer Chemo-resistance and Inhibits Tumour Development in TRAMP Mice. *Sci. Rep.* **6**, 32062; doi: 10.1038/srep32062 (2016).

## Supplementary Material

Supplementary Information

## Figures and Tables

**Table 1:** Parametric blood composition analysis for TRAMP mice.

S. No	Blood Parameter	Control	Dox IP	Fe-bLf-Dox Feed
1	WBC (103/mm^3^)	2.366 ± 1.307	2.966 ± 0.555	5.166 ± 1.108
2	RBC (106/mm^3^)	6.01 ± 3.489	2.47 ± 1.001 (L)	9.52 ± 0.499
3	HGB (g/dL)	10 ± 5.256	5.633 ± 1.184 (L)	15.333 ± 0.984
4	HCT (%)	28.9 ± 17.54 (L)	10.53 ± 4.733 (L)	44.33 ± 3.445
5	MCV (μm^3^)	46.33 ± 3.091	41.67 ± 3.299	46.333 ± 1.247
6	MCH (pg)	17.66 ± 1.800	25.23 ± 8.017 (H)	16.066 ± 0.34
7	MCHC (g/dL)	38.56 ± 6.86	62.43 ± 25.17 (H)	34.63 ± 1.319
8	PLT (103/mm^3^)	136 ± 27.796	634 ± 265.92	189.33 ± 66.10
9	Serum Iron (μg/dL)	183.33 ± 10.338	121 ± 12.832	239.33 ± 26.83

WBC - white blood cells, RBC - red blood cells, HGB - haemoglobin, HCT - haematocrit, MCV - mean corpuscular volume, MCH - mean corpuscular haemoglobin, MCHC -mean corpuscular haemoglobin concentration, PLT-platelets. Values lower than the normal levels are indicated (L) and values higher are indicated (H).
