# Author Correction: Doxorubicin Conjugated to Immunomodulatory Anticancer Lactoferrin Displays Improved Cytotoxicity Overcoming Prostate Cancer Chemo resistance and Inhibits Tumour Development in TRAMP Mice

**DOI:** 10.1038/s41598-019-42459-5

**Published:** 2019-04-25

**Authors:** Jayanth Suryanarayanan Shankaranarayanan, Jagat R. Kanwar, Afrah Jalil Abd AL-Juhaishi, Rupinder K. Kanwar

**Affiliations:** Nanomedicine-Laboratory of Immunology and Molecular Biomedical Research, School of Medicine, Faculty of Health, C-MMR, Deakin University, Geelong, Victoria 3216 Australia

Correction to: *Scientific Reports* 10.1038/srep32062, published online 31 August 2016

This Article contains errors.

In Figure 3C, the merge images of confocal micrographs for Apo-bLf 10 nM and Dox 1.5 µM are incorrect and represents a different replicate of same treatment group.

Due to an error that occurred at the time of figure assembly, the representative image for 48 h Fe-bLf sample in Figure 5F was included as a duplicate image of 96 h Apo-bLf sample. In addition, for 48 h untreated sample, the representative image is incorrect and shows a different sample.

The correct versions of Figures 3C and 5F are given below as Figures 1 and 2.

**Figure 1:**
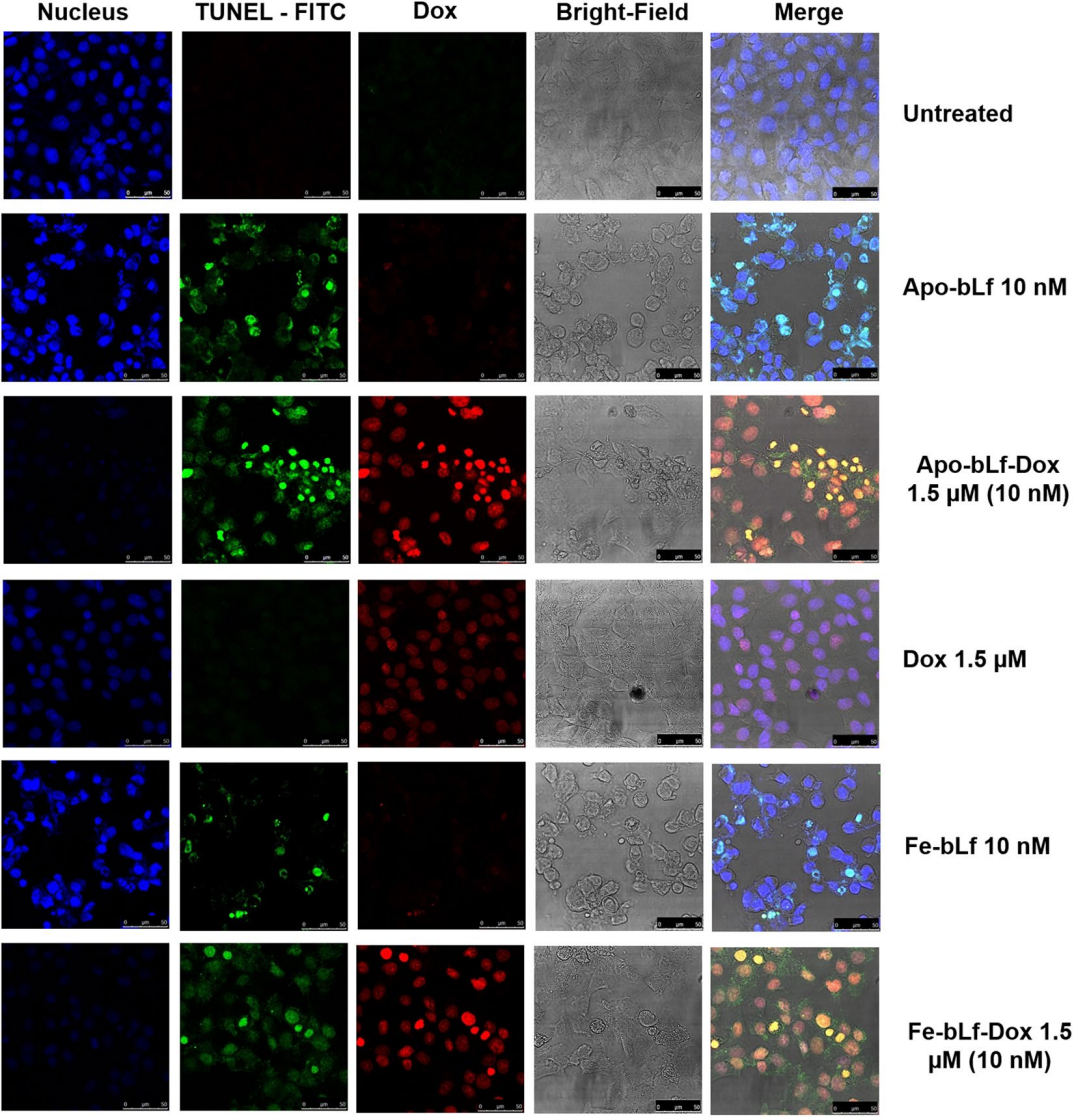
bLf-Dox conjugates treatment induces cancer cell apoptosis. (**C**) The presence of fragmented DNA as an end product of apoptotic cascade within the cell was considered as the confirmatory test for induction of apoptosis especially the Dox mediated DNA damage using TUNEL assay. The nucleus was counterstained with DAPI and Dox auto-fluorescence is represented in red.

**Figure 2:**
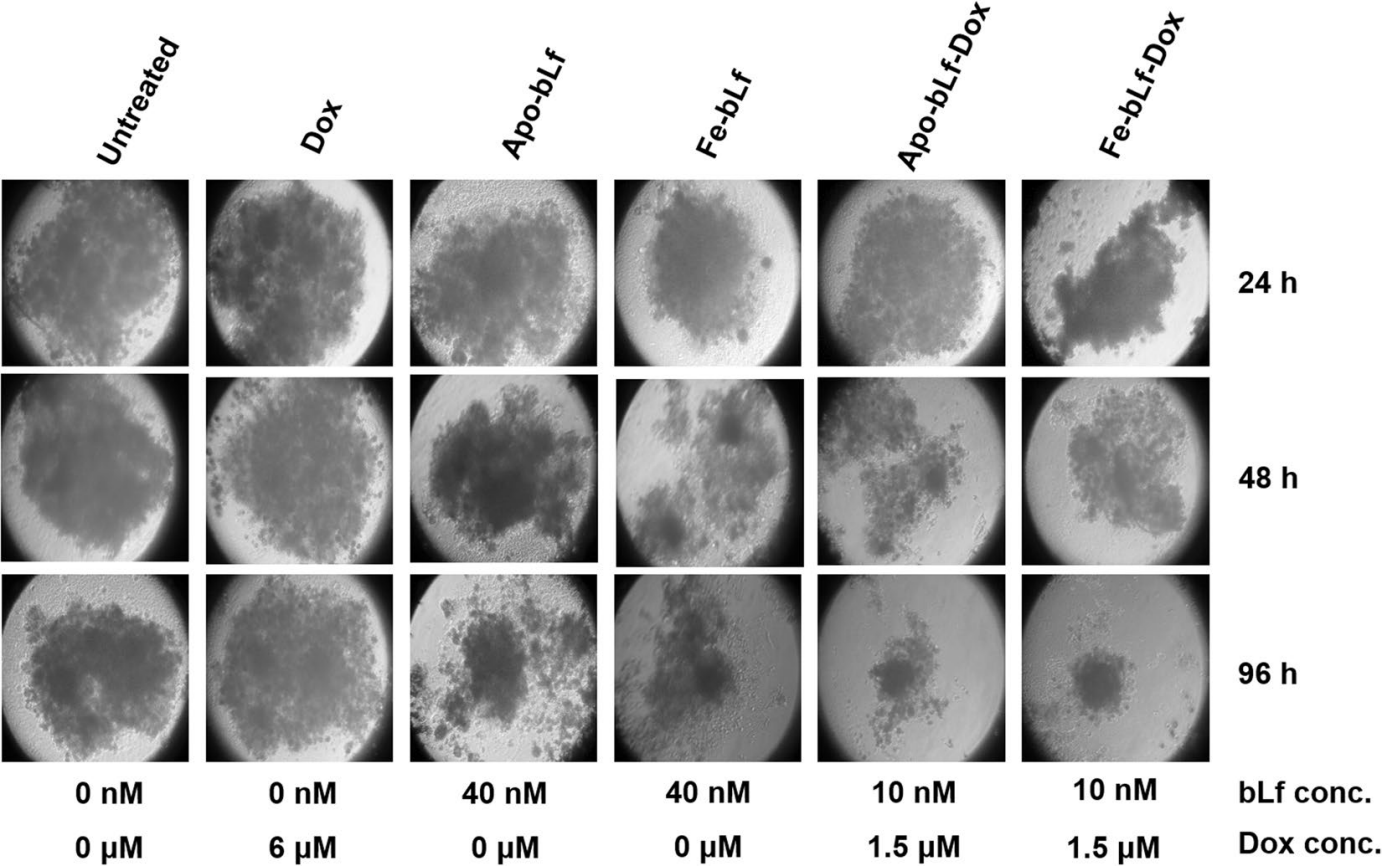
Advanced drug resistant (ADR1000-DU145) cells were sensitive to bLf-Dox treatments but not Dox alone. (**F**) ADR1000-DU145 cells were allowed to form prostaspheres for 7 days. Following spheroid formation, they were treated once at 0 h and again at 48 h and the spheroids were imaged under the microscope.

This change does not affect the conclusions of the Article. The authors apologize for the errors.

