# Supplementary material for: Doxorubicin Conjugated to Immunomodulatory Anticancer Lactoferrin Displays Improved Cytotoxicity Overcoming Prostate Cancer Chemo resistance and Inhibits Tumour Development in TRAMP Mice
Source: Sci Rep. 2016 Aug 31;6:32062. doi: 10.1038/srep32062 (PMC5005995; doi:10.1038/srep32062)
Supplement: Supplementary Information [file srep32062-s1.pdf]

Doxorubicin Conjugated to Immunomodulatory Anticancer Lactoferrin Displays Improved Cytotoxicity Overcoming Prostate Cancer Chemoresistance and Inhibits Tumour Development in TRAMP Mice

Jayanth Suryanarayanan Shankaranarayanan, Jagat R. Kanwar, Afrah Jalil Abd AL-Juhaishi, Rupinder K. Kanwar**

Nanomedicine-Laboratory of Immunology and Molecular Biomedical Research, School of Medicine, Faculty of Health, C-MMR, Deakin University, Geelong, Victoria 3216, Australia.

AUTHOR INFORMATION

***Corresponding Author**

Dr. Rupinder K Kanwar

Nanomedicine-Laboratory of Immunology and Molecular Biomedical Research, School of Medicine, Faculty of Health, Deakin University, Geelong, Victoria 3216, Australia.

Phone: +61-3-5227 2420

Email – rupinder.kanwar@deakin.edu.au

Co-Corresponding Author

Professor. Jagat R Kanwar

Nanomedicine-Laboratory of Immunology and Molecular Biomedical Research, School of Medicine, Faculty of Health, Deakin University, Geelong, Victoria 3216, Australia.

Phone: +61-3-52271128

Email: jagat.kanwar@deakin.edu.au

Supplementary Information

1.1 Supplementary Methods

Sorting of cells for cancer stem cells

Isolation and enrichment of stem cell populations in advanced prostate cancer cell line of DU145 was carried out using magnetic bead based separation technique. In this technique, cancer stem cells were specifically labelled for the expression of stem cell markers such as EpCAM and CD44 using antibodies linked to magnetic beads and were separated using the magnetic columns (MACS® Miltenyi Biotec, Germany) according to manufacturer's instruction. CD44+ and EpCAM+ double positive DU145 cells were thus obtained by enriching the cells with EpCAM positive cells using first round of separation which were then re-enriched for CD44 positive population. The sorted cells were analysed by flow cytometry for expression of respective stem cell markers.

Cell lysis and isolation of total protein for Western blot analysis of protein expression changes

DU145 cells were plated out in a 6 well plate at a density of $2x10^5$ cells/mL and incubated at 37 °C, 5% CO_2 and were allowed to grow overnight. Once confluent, the cells were treated with different concentrations of Dox alone or the bLf-Dox conjugates. The cells were then incubated with the treatments for 24 h at 37 °C at 5% CO_2. After incubation with the treatments, the supernatant was discarded and the cell layer was washed with PBS. 100 µL of RIPA buffer containing 1x protease inhibitor was added to each well and the plate was placed on ice for 20 min [68]. The cell layer was scraped using a sterile cell scrapper and collected in microcentrifuge tubes. The lysates were first sonicated at 75% amplitude for 30 s in ice to dislodge any membrane bound proteins and were centrifuged at 13,000 rpm for 20 min at 4 °C. The supernatants were collected after centrifugation and stored in a fresh microcentrifuge tube. Samples were then measured for their protein concentration using Bradford's reagent. 100 µg of total cell lysate was loaded for each treatment and Western blotting was carried out using standard protocol with appropriate primary and secondary antibodies.

Western Blotting

The transfer of proteins from the poly acrylamide gel to a PVDF membrane (GE Biosciences, Australia) was carried out on a Trans-Blot Turbo semi dry transfer system (Bio-Rad, Australia). After the blocking process, the membrane was incubated with the primary antibodies at a 1:1000 dilution in TBS for 1 h at 37 °C. Following incubation, the membrane was washed with TBS-T and incubated with respective HRP

conjugated secondary antibody (Sigma-Aldrich, Australia) at 1:10,000 for 1 h at 37 °C. The washes were repeated and finally the membrane was developed using HRP substrate based ECL Chemi-luminescence reagents (GE Biosciences, Australia); the membrane was imaged under dark using Chemi-Doc XRS+.

TUNEL assay

DNA fragmentation post treatments were evaluated using Terminal Deoxynucleotidyl Transferase dUTP Nick End Labelling [TUNEL] kit. $1X10^4$ cells were seeded on an 8 well slide and allowed to grow until they attained 90% of confluency and cells were treated accordingly. Untreated cells were taken as controls along with a negative control containing only the fluorescent nucleotides without the enzyme. After treatment, cells were washed with PBS and permeabilised with 0.1% Triton X-100 and resuspended in 50 µL of TUNEL reaction (Containing the transferase enzyme and the FITC labelled nucleotides) mixture. The cells were incubated for about 60 min at 37 °C in dark. The slides were then counter-stained for the nucleus and imaged under the confocal microscope.

Clonogenic assay

ADR1000-DU145 cells were plated out in a 6 well plate and treated with appropriate treatments for 24 h. After this, the treatment was removed and the cells were trypsinised. About 200 cells were counted from each treatment and plated out as single cell suspension in a 6 well plate. The cells were allowed to grow for 10-14 days to allow them to form colonies. After that, the cells were fixed using paraformaldehyde and stained with 1% crystal violet to visualise the colonies which were then counted manually using an illuminated colony counter. Representative photographs were taken for each treatment.

Migration assay

Transwell membrane Boyden chambers (Thin-certs) with a pore size of 8 µm were placed in a 24 well plate and 1mL of 10^5 cells (ADR1000-DU145) were plated out in the top chambers with 2 mL of media in the bottom chamber [66]. To the wells, media with varying treatment concentrations were added including one well as a control with media alone. Cells were incubated with treatments for 24 h at 37 °C with 5% CO_2. Cells were then fixed with 4% PF for 20 min at room temperature following which they were washed with PBS and then stained with 0.1% crystal violet. The non-migrated top layer of cells was removed with the help of a cotton. The cells were then viewed, counted and imaged using an inverted microscope.

Immunohistochemical analysis

Following dissection, brain and small intestine from all groups were fixed in 4% freshly prepared paraformaldehyde (in PBS pH 7.4) for 18 h. Samples were then washed, dehydrated, and embedded in paraffin using Leica Paraffin Embedding Sample Prep Processor and Paraffin Embedding Station. Sections of 7µm thickness were stained either with Hematoxylin and Eosin (H&E) for histological observation. Demonstration of CCL11 immunostaining in brain and intestine sections of normal and treated mice was carried out by deparaffinizing the sections and incubation with 5µg/mL of goat polyclonal anti-mouse CCL11 antibody from R&D systems (AF-420-NA), for one hour at 37^0 C. The primary antibody binding was detected using R&D Systems Anti-Goat HRP-DAB Cell & Tissue Staining Kit protocol as per manufacturer's instructions. The immunoreactivity in terms of color intensity detected with (3, 3-diaminobenzidine tetrahydrochloride DAB) was visualized and imaged with the Leica bright field microscope. A total of 10 microscopic fields for each section were visualized for immunopositive zones of the tissue.

1.2 Supplementary Results

Fe-bLf-Dox conjugates were less cytotoxic to non-cancerous cells compared to the Dox alone treatment

In comparison to the cytotoxic Dox alone treatment, the Apo-bLf-Dox and Fe-bLf-Dox showed lower toxicity (P<0.001) in the lower concentration range of 1.5 µM and 3 µM. At 3 µM concentration Apo-bLf-Dox induced a 43.8% cytotoxicity which was significantly higher (P<0.001) than the 14.2% cytotoxicity induced by Fe-bLf-Dox at the same concentration, whereas it was much lower than the 81.5% cytotoxicity induced by Dox alone at the 3 µM concentration (**Supplementary Information**). At the high concentration of 6 µM there was no significant difference between the cytotoxicity of Dox alone and Apo-bLf-Dox, but, the cytotoxicityof Fe-bLf-Dox (55.32%) was significantly lesser than either of Apo-bLf-Dox and Dox alone treatments (P<0.001).

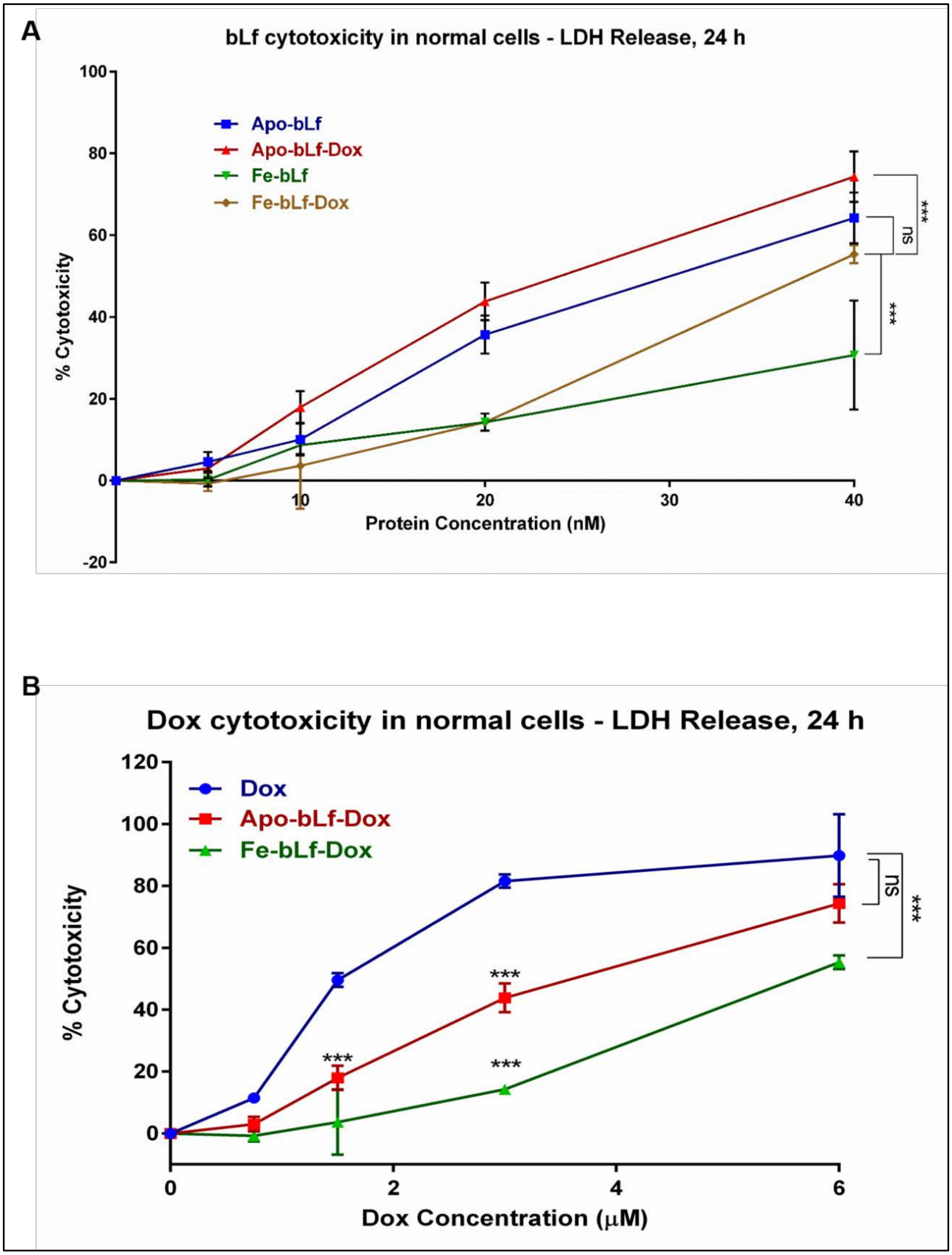

Figure S.1: bLf-Dox conjugates inflicted lower cytotoxicity on non-cancerous RWPE-1 cells in comparison to Dox alone

bLf-Dox conjugates retain their anti-cancer effectiveness against stem cell enriched in DU145 $CD44^+$/$EpCAM^+$ double positive cells

DU145 $CD44^+$/$EpCAM^+$ double positive cells represented a stem cell enriched highly aggressive sub-population of DU145 cells which are considered responsible for tumour relapse and chemoresistance. The effectiveness of bLf-Dox conjugates to reduce the clonogenic potential of DU145 CD44+/EpCAM+ double positive was also observed. It can be seen from **Figure S.2 A and B** that all the treatments reduced the number of colonies formed significantly (P<0.001). Dox at 6 μM concentration was surprisingly very effective. However, the most effective treatments were that of Apo-bLf-Dox and Fe-bLf-Dox which at 1.5 μM Dox equivalents completely inhibited the clonogenic property at a 4 fold lower concentration which is considered a marked improvement in the activity of both bLf as well as Dox. The qualitative and quantitative analysis of number of migrated cells following treatment (**Figure S.2 C and D**) also showed that Apo-bLf and Fe-bLf (40 nM) significantly inhibited the migration (P<0.001) of these DU145 CD44+/EpCAM+ double positive cells. These results exemplified that Dox at 6 μM was capable of reducing the migratory potential however, was not as effective as the conjugates at 1.5 μM Dox equivalent concentration.

The successful results from the 2D culture studies were then extrapolated to be studied in 3D culture. It can be seen from the images of the prostaspheres (**Figure S.2 E**) that there was a gradual decrease in their size over time. Fe-bLf-Dox and Apo-bLf-Dox showed the highest reduction in spheroid diameter (P<0.001) after 24 h, 48 h and 96 h incubation periods. In the 96 h treatment of the bLf-conjugates the spheroids were almost completely vanished. Both Fe-bLf and Apo-bLf when given alone showed significant reduction in 24 h and 48 h (P<0.05) and a highly significant reduction in 96 h following a second dose after 48 h (P<0.001). Dox on the other hand showed a decrease during the first 24 h, however, a significant decrease was observed only after the 96 h period involving two doses (P<0.001). Trypan blue analysis (**Figure S.2 G**) performed on the cells obtained after 96 h treatment confirmed the above results.

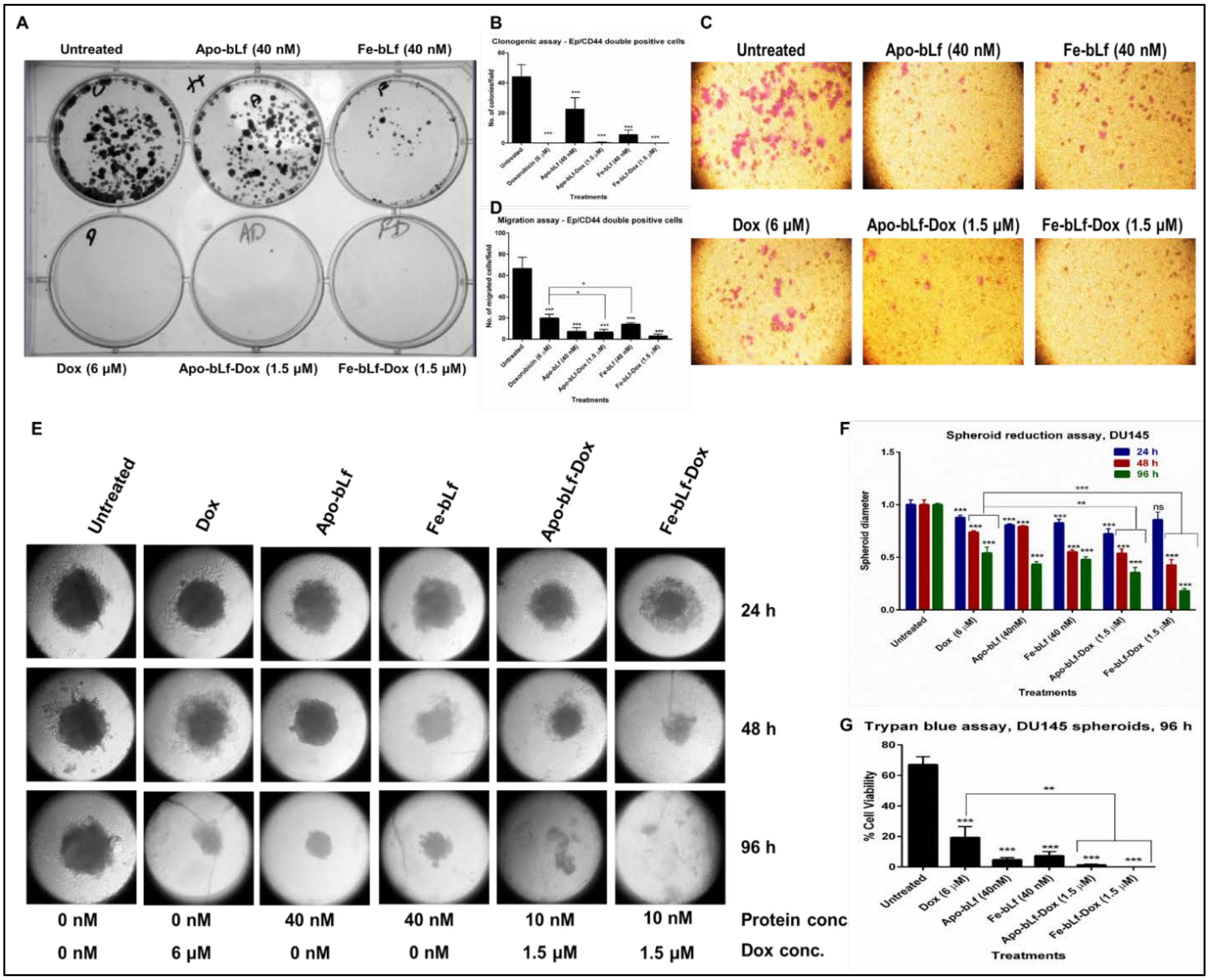

Figure S.2: bLf-Dox conjugates reduced the aggressiveness of stem cell enriched CD44+/EpCAM+ double positive cells

A.) The colony formation and survival assay was used to determine the ability of single DU145 CD44+/EpCAM+ double positive cells to replicate and form colonies following exposure to treatments.

B.) Colonies were manually counted under colony counter and the numbers of colonies formed per field of analysis are given in the form of a histogram. Treatments were carried out in triplicates and the assay was performed thrice independently. Data has been expressed as mean ± SD. One-way ANOVA was performed to evaluate statistical significance followed by post-hoc analysis by Tukey's test.

C.) The transwell chamber migration assay was used to determine the ability of DU145 CD44+/EpCAM+ double positive cells to migrate through an 8 micron transwell thincerts following exposure to treatments. The migrated cells were then stained with 0.1% cyrstal violet and imaged.

D.) Migrated cells were manually counted under a microscope and the numbers of colonies formed per field of analysis are given in the form of a histogram. Treatments were carried out in triplicates and the assay was performed thrice independently. Data has

been expressed as mean ± SD. One-way ANOVA was performed to evaluate statistical significance followed by post-hoc analysis by Tukey's test.

E.) DU145 CD44+/EpCAM+ double positive cells were allowed to form prostaspheres for 7 days. Following spheroid formation, they were treated once at 0 h and again at 48 h and the spheroids were imaged under the microscope.

F.) The size (Diameter) of the tumour spheroid was measured at 24 h, 48 h and 96 h post first treatment was measured using Image J (NIH) software tool. The fold change in the reduction of spheroid diameter was calculated and represented as histogram. The experiment was carried out thrice with 5 spheroids per treatment. Two-way ANOVA was performed to evaluate statistical significance followed by post-hoc analysis by Tukey's test.

G.) Trypan blue analysis was performed post 96 h treatment to analyse the percentage viable cells left in the spheroids.

Dox induces neurotoxicity, upregulates CCL11 in mice brain while Fe-bLf-Dox conjugate is neuroprotective and inhibits CCL11 expression

We performed immunohistochemical analysis to determine, if the significantly high serum presence of CCL11 could have induced adverse actions in the brain tissue of Fe-bLf- Dox treated mice. The elevated circulating CCL11 can contribute to cognitive decline in aging by directly inhibiting adult hippocampal neurogenesis[1]. The mechanism by which circulating CCL11 induce neurodegeneration is not clear. Recently, a study has reported that CCL11 crosses the BBB (blood brain barrier), its transport occurs throughout the brain with varied transport rates among brain regions, and transport is affected by interactions with the cellular components of blood. The authors suggests that circulating CCL11 levels can be regulated by the BBB and could therefore effect its actions in the CNS[2].

On the other hand, Dox is known for its neurotoxicity and neuron damaging effects due to induction of oxidative stress and ROS generation[3]. As shown in **Figure S.3 A and B**, Dox treated mice brain tissue sections in addition to upregulated CCL11 Expression, showed eosinophil infiltration, and loss of neuronal tissue and architecture. Fe-bLf-Dox treatment (**Figure S.3 D**) restored very well the tissue architecture and no CCL11 expression was observed, though untreated mice brain tissue showed very low CCL11 expression.

The lack of CCL11 expression in Fe-bLf-Dox fed mice brain despite its significantly higher concentration in serum than the Dox (i.p.) treated animals, is intriguing. For its anti-inflammatory activity, lactoferrin (a glycosaminoglycan-binding molecule) has been shown to compete with cytokines and chemokines for its binding to proteoglycans. For example, it binds with a very high affinity than IL-8, to endothelial glycosaminoglycans involved in chemotaxis[4]. Thus it could be possible that the transport of serum CCL11 to BBB was either competitively inhibited by Fe-bLf-Dox conjugate through its binding to proteoglycans on BBB endothelia or Fe-bLf's binding to Lf receptors present on BBB and blood cells. As mentioned above, circulating CCL11 transport to brain regions is affected by interactions with the cellular components of blood[2]. An interesting future research can be considered based on our current findings.

The observations of well-preserved brain tissue architecture with Fe-bLf-Dox fed animals, could be explained in terms of the neuroprotective and antioxidant nature of bLf molecule which is gaining scientific attention, recently. We have reported that Fe-bLf involved both the PI3K and ERK signaling for inducing neuronal differentiation[5], while bLf's neuroprotective function in preventing prion protein-induced cell death, as an antioxidant due to the scavenging of ROS has also been reported[6]. Human lactoferrin (hLf) protects vulnerable dopamine neurons from degeneration in Parkinson's disease by preserving calcium homeostasis in mitochondria[7].Further the efficacy of (hLf) was comparable to that of glial cell line–derived neurotropic factor (a prototypical neurotrophic factor for dopamine neurons. The retained neuroprotective ability of Fe-bLf, while fed orally as a protein-drug conjugate to circumvent Dox induced neurotoxicity, is promising and warrants further research to delineate the exact mechanism which is beyond the scope of current study.

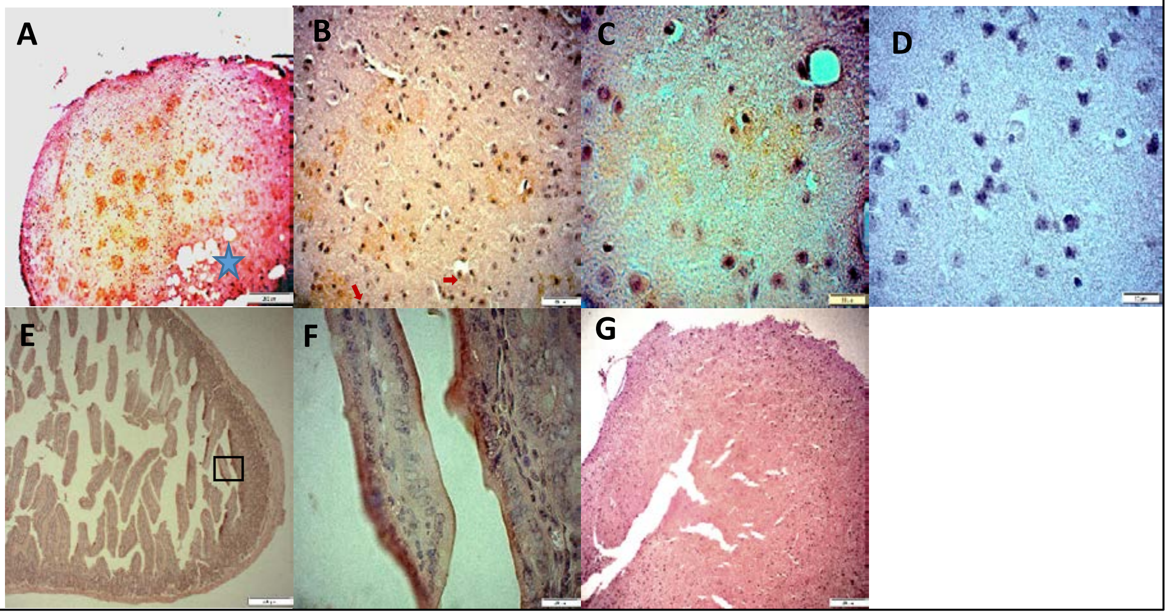

Figure S-3: **Representative bright field microscopy images of paraffin embedded mice tissue sections stained with anti-mouse CCL11 /Eotaxin antibody (R&D AF-420-NA). The primary antibody binding was detected using R&D Systems Anti-Goat HRP-DAB Cell & Tissue Staining Kit**.

A & B) CCL11 expression within doxorubicin (I.P.) treated mice brain tissue. Blue Star shows neuronal loss area and neurotoxicity of Dox. Blue star in **A** indicates loss of brain tissue architecture and neuronal loss region of brain. Red arrows in **B** show the eosinophil infiltration.

C) Control group mice brain reveals patchy and very low CCL11 expression.

D) Fe-bLf-Dox conjugate treated mice brain tissue revealed no CCL11 expression, and normal brain architecture was observed

E & F) Positive control illustrates strong expression of CCL11 within the intestinal villi. **G)** No expression for CCL11 was seen in primary antibody control.

Scale bar= 50 μM (A, D & F), = 200 μM (B, C, E and G).

References

1. Villeda, S.A, et al. The ageing systemic milieu negatively regulates neurogenesis and cognitive function. *Nature* **477**: 90–94 (2011).

2. Erickson, M. A., et al. "Rapid transport of CCL11 across the blood-brain barrier: regional variation and importance of blood cells." *The Journal of Pharmacology and Experimental Therapeutics* **349**: 497-507. (2014).

3. Sankhadeep, P, Ahir, M., & Parames, C. S. Doxorubicin induced neurotoxicity is attenuated by a 43-kD protein from the leaves of *Cajanus indicus L.* via NF-κB and mitochondria dependent pathways. *Free Radical Research* **46**: 785-798 (2012).

4. Elass, E., Masson, M., Mazurier, J., & Legrand, D. Lactoferrin Inhibits the Lipopolysaccharide-Induced Expression and Proteoglycan-Binding Ability of Interleukin-8 in Human Endothelial Cells. *Infection and Immunity*, **70**, 1860–1866 (2002).

5. Sriramoju, B., Kanwar, R.K., &Kanwar, J.R. Lactoferrin induced neuronal differentiation: A boon for brain tumours." *International Journal of Developmental Neuroscience* **41**: 28-36. (2015).

6. Park, Y. G., et al. Lactoferrin protects against prion protein-induced cell death in neuronal cells by preventing mitochondrial dysfunction. *International Journal of Molecular Medicine* **31**: 325-330 (2013).

7. Rousseau, E., Patrick P.M., and Etienne C. H. The iron-binding protein lactoferrin protects vulnerable dopamine neurons from degeneration by preserving mitochondrial calcium homeostasis. *Molecular Pharmacology* **84**: 888-898 (2013).
